# Optimal energy and redox metabolism in the cyanobacterium *Synechocystis* sp. PCC 6803

**DOI:** 10.1038/s41540-023-00307-3

**Published:** 2023-09-22

**Authors:** Amit Kugler, Karin Stensjö

**Affiliations:** Microbial Chemistry, Department of Chemistry-Ångström Laboratory, Uppsala University, Box 523, SE-751 20 Uppsala, Sweden

**Keywords:** Computer modelling, Biochemical networks, Biosynthesis

## Abstract

Understanding energy and redox homeostasis and carbon partitioning is crucial for systems metabolic engineering of cell factories. Carbon metabolism alone cannot achieve maximal accumulation of metabolites in production hosts, since an efficient production of target molecules requires energy and redox balance, in addition to carbon flow. The interplay between cofactor regeneration and heterologous production in photosynthetic microorganisms is not fully explored. To investigate the optimality of energy and redox metabolism, while overproducing alkenes—isobutene, isoprene, ethylene and 1-undecene, in the cyanobacterium *Synechocystis* sp. PCC 6803, we applied stoichiometric metabolic modelling. Our network-wide analysis indicates that the rate of NAD(P)H regeneration, rather than of ATP, controls ATP/NADPH ratio, and thereby bioproduction. The simulation also implies that energy and redox balance is interconnected with carbon and nitrogen metabolism. Furthermore, we show that an auxiliary pathway, composed of serine, one-carbon and glycine metabolism, supports cellular redox homeostasis and ATP cycling. The study revealed non-intuitive metabolic pathways required to enhance alkene production, which are mainly driven by a few key reactions carrying a high flux. We envision that the presented comparative in-silico metabolic analysis will guide the rational design of *Synechocystis* as a photobiological production platform of target chemicals.

## Introduction

Cyanobacteria are photosynthetic microbes that serve as an attractive platform for the sustainable production of chemicals and fuels, mainly due to their capability of converting atmospheric carbon dioxide into organic compounds by using solar energy, their relative rapid growth rate, and the readily available genetic toolbox for various species^1^. In the past years, the unicellular cyanobacterium *Synechocystis* sp. PCC 6803 (hereafter *Synechocystis*) has been successfully used as a cell factory for the production of commodity chemicals, including, *n*-butanol^2^, bisabolene^3^ and phenylpropanoids^4^. Besides autotrophy, cyanobacteria are able to grow under mixotrophic conditions. Mixotrophic metabolism allows the cells to catabolize organic carbon parallel to CO_2_ being assimilated photosynthetically. Addition of organic substrates in light growth conditions, though increasing the risk of contamination and cost, is considered as a promising cultivation strategy for the commercialization of cyanobacteria^5,6^, owing to enhanced biomass^7^ and chemical yield^8,9^.

The biosynthesis of various cell metabolites involves biochemical reactions, which require cofactors, such as adenosine triphosphate (ATP) and reduced nicotinamide adenine dinucleotides [NAD(P)H]. ATP is the major energy carrier in the cell, driving numerous metabolic pathways. The pyridine nucleotides NADH and NADPH serve as reducing equivalents in redox reactions. While NADH participates mainly in catabolic reactions, NADPH plays a role in anabolic reactions. In cyanobacteria, the photosynthetic electron transport chain is the major source for ATP and NADPH generation, when grown under light conditions^10^. The different glycolytic routes and the TCA cycle generate reducing agent in the form of NADH. In addition, transhydrogenases catalyze the interconversion between NADH and NADPH^11^.

Optimal performance of photosynthesis requires that the light-energy conversion by the photosystems and the downstream metabolic pathways are fine-tuned, to support the ATP/NADPH output ratio and the cellular energy economy^12^. The photosynthetic linear electron flow (LEF) in *Synechocystis* generates an ATP/NADPH ratio of approximately 1.28, which is less than required for CO_2_ fixation by the Calvin–Benson–Bassham (CBB) cycle and downstream biochemical pathways^12^. To deal with this imbalance, alternative pathways contributing to ATP production exist^13,14^. In addition, re-oxidation of NAD(P)H to NAD(P)^+^ and phosphorylation-dephosphorylation of reducing equivalents are important for balancing the ATP/NADPH budget and maintaining redox homeostasis^15^. Despite the importance of meeting the energy and redox requirement to achieve an efficient cellular performance^16,17^, only few metabolic engineering efforts of *Synechocystis* have targeted this designing strategy^18^, while most studies are concerned with controlling carbon partitioning^1^.

Constraint-based modelling (CBM) has been employed for the characterization of physiological capabilities of *Synechocystis*, for which several detailed genome-scale metabolic reconstructions are available^19–25^. CBM is based on imposing of a set of constraints that govern the operation of a metabolic network at steady state. These include, for example, the stoichiometry of the biochemical reactions, mass balance and thermodynamic laws. Flux balance analysis (FBA) is a common CBM-based approach to calculate, under given constraints, the intracellular flux distributions within the stoichiometric network, by optimizing an objective function^26^. As such, FBA can be used to predict the maximum (or minimum) growth rate or the production of desired metabolites, as well as to identify genetic interventions that force carbon flux toward chosen compound. Case studies demonstrating model-driven metabolic engineering of *Synechocystis* include the overproduction production of isoprene^27^, *n*-butanol^28^ and ethanol^29^.

In this work, we focus on alkenes, commercially valuable platform chemicals, which are traditionally used as detergents, lubricants and rubbers. In addition, they are compatible hydrocarbon fuels, due to their high energy content^30^. The development of gas-to-liquid technologies, enabling the oligomerization of small gaseous substrates into liquid chemicals, together with the environmental concerns associated with the use of fossil fuels and carbon dioxide emissions^31^, are driving the necessity for an environmentally-friendly, yet economically-feasible, manufacturing process of bio-alkenes^32,33^.

We addressed the question of how the model cyanobacterium *Synechocystis* balances its anabolic-catabolic processes, with respect to central cofactor metabolites [ATP and NAD(P)H], during growth under autotrophic and mixotrophic conditions. To this end, we employed genome-scale metabolic modelling, and analyzed the metabolism of *Synechocystis* strains overproducing alkenes as a case study.

## Results and discussion

**Fig. 1:**
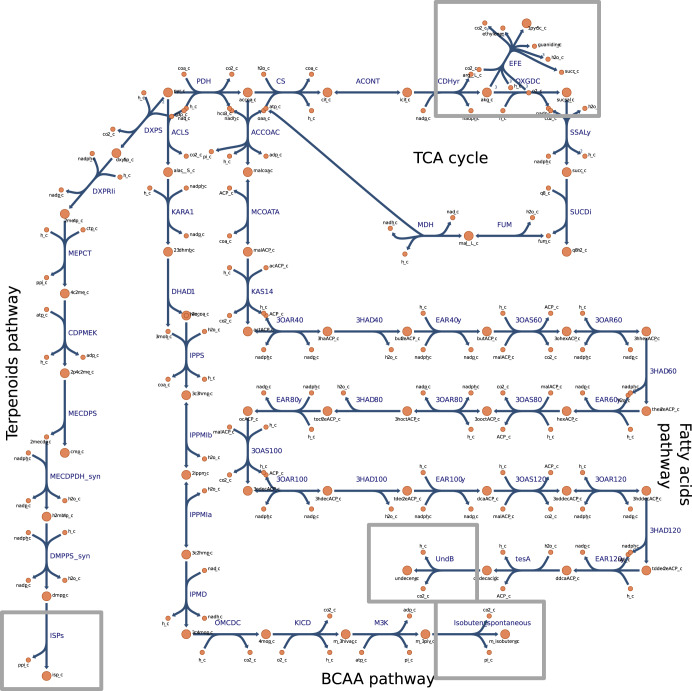
Non-native alkene biosynthesis pathways reconstructed in the iJN678_AK *Synechocystis* sp. PCC 6803 genome-scale model for cofactor balance analysis. TCA tricarboxylic acid, BCAA branched-chain amino acids. Irreversible reactions are indicated by one-headed arrows; reversible reactions are indicated by two-headed arrows. Metabolic reactions and metabolites (except heterologous ones) are indicated by their BiGG identifier^73^.

To enable a system analysis of alkene-overproducing *Synechocystis* strains, and assess the resulting energy and redox (im)balance, we chose four alkenes whose production using cyanobacteria has been previously demonstrated: isoprene^34^, isobutene^35^, ethylene^36^ and 1-undecene^37^. The four alkenes stem from different metabolic routes and possess different cofactors requirements (Fig. 1, Table 1).

**Table 1:** Evaluated turnover rates of energy and redox carriers.

	ATP		NADPH		NADH		ATP/NADPH	
Objective	Auto	Mixo	Auto	Mixo	Auto	Mixo	Auto	Mixo
Biomass	8.15	5.35	3.87	0.28	0.12	0.07	2.11	19.49
Isoprene	8.36	5.25	5.09	0.93	0.01	0.01	1.64	5.67
Isobutene	8.61	5.81	5.47	1.85	0.35	0.26	1.57	3.15
Ethylene	7.67	6.82	4.26	2.68	0.49	0.55	1.80	2.55
1-undecene	7.24	5.38	5.49	1.19	0.01	0.01	1.32	4.51

*Synechocystis* was simulated to grow autotrophically and mixotrophically, when maximizing biomass and alkenes production. For each end-product, the ATP, NADPH and NADH turnover rates and the corresponding ATP/NADPH ratios were computed.

*Auto* autotrophic, *Mixo* mixotrophic.

### Evaluation of maximal production capability

**Fig. 2:**
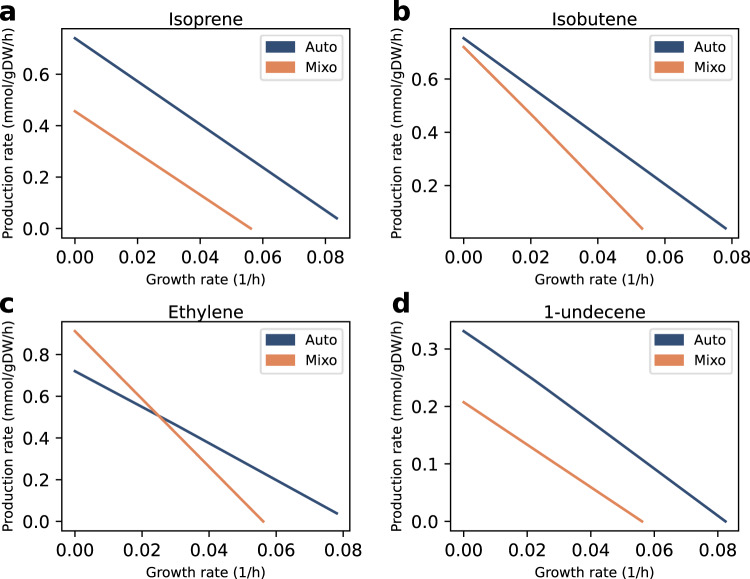
Alkene-biomass phenotypic phase planes. **a** Isoprene. **b** Isobutene. **c** Ethylene. **d** 1-undecene. Phenotypic phase planes were obtained by varying these fluxes in the range from zero to maximal rate, under constant photon flux. Auto autotrophic, Mixo mixotrophic.

**Fig. 3:**
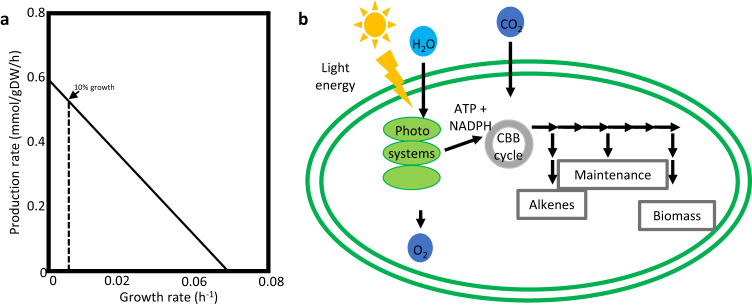
Factors influencing the stoichiometric evaluation in this study. **a** As a trade-off occurs between biomass and alkene production, a two-step optimization strategy was performed, as described in the Methods section. **b** Cellular resources are allocated to biomass, cell maintenance and alkene production.

First, we examined how increasing growth rate affected alkene production, using phenotypic phase plane analysis^38^. Under both autotrophic and mixotrophic growth conditions, we noticed a trade-off between growth (biomass production) and product synthesis. Predicted maximal alkene production rates resulted in zero biomass accumulation, and vice-versa (Fig. 2a–d). Therefore, we aimed to simulate a more real scenario, in which a cell produces high amounts of alkenes, whilst sustaining minimal biomass synthesis (Fig. 3a). We performed the computational analysis in a two-step optimization strategy (see Methods section).

Subsequently, we evaluated the theoretical maximum production capability, by analyzing the maximum productivity, maximum mass yield and maximum c-mol yield (Table 2); all are parameters of interest from both biotechnological and biochemical aspects. By comparing carbon-source-dependent bioproduction, our computational analysis illustrated that the maximum theoretical mass yield is approximately doubled under mixotrophic conditions, as compared to autotrophic condition, for the production of the examined alkenes. Except for 1-undecene, autotrophic conditions resulted in a comparable productivity for all objectives. Under both growth conditions, the yields were similar for all alkenes, except for ethylene, for which these values were lower. Under autotrophic growth, the release rate of CO_2_ is 3.81 mmol/gDW/h for biomass production, whereas ethylene production resulted in a release rate of 5.27 mmol/gDW/h (Supplementary Table 6). Thus, the low yields found for ethylene can be owed to the high carbon loss.

**Table 2:** Theoretical maximum productivity, mass yield and c-mol yield.

	Auto			Mixo		
Objective	Maximum productivity	Maximum theoretical mass yield	C-mol yield	Maximum productivity	Maximum theoretical mass yield	C-mol yield
Biomass	0.082			0.056		
Isoprene	0.67	0.20	0.90	0.41	0.41	0.90
Isobutene	0.68	0.20	0.87	0.51	0.42	0.90
Ethylene	0.65	0.15	0.65	0.82	0.34	0.73
1-undecene	0.30	0.21	0.91	0.19	0.42	0.90

*Synechocystis* was simulated to grow autotrophically and mixotrophically, when maximizing biomass and alkenes production. Maximum productivity is presented in units of h^−1^ or mmol/gDW/h, for biomass and alkenes, respectively. Mass yield is presented in units of gram product per gram substrate. Biomass yield is represented in units of gDW biomass per mmol carbon consumed. C-mol yield is presented in units of mol carbon produced per mol carbon consumed.

*gDW* gram dry-weight, *h* hour, *C* carbon, *Auto* autotrophic, *Mixo* mixotrophic.

### Evaluation of ATP and NAD(P)H demands

We then analyzed *Synechocystis* metabolism from the metabolite-centric point of view, also termed as flux-sum analysis^39^. This approach is useful for understanding metabolite essentiality^40^, and for studying the intracellular network robustness to perturbations in metabolite turnover rates^39^. Even though the net accumulation of a metabolite might be zero, the overall turnover rate is an indicator of the importance of the specific metabolite.

Under both autotrophic and mixotrophic growth, a higher usage of NADPH (turnover rate of 3.87–5.49 mmol/gDW/h), and ATP (turnover rate of 7.24–8.61 mmol/gDW/h) was observed, as compare to the usage of NADH (turnover rate of 0.01–0.49 mmol/gDW/h) (Table 1). These results are in line with previous experimental studies, showing that NADPH is being preferentially used over NADH in *Synechocystis*, when grown under autotrophic conditions^10^. In addition, all strains exhibited a comparable ATP requirement, although with some variations. For example, less ATP was required for 1-undecene production under autotrophic conditions, and for isoprene under mixotrophic conditions. Under mixotrophic conditions, lower turnover rates were observed for both NADPH and NADH. An explanation for these observations is that the introduced biosynthesis pathways are mainly anabolic, and therefore use more NADPH than NADH.

Since the overall consumption and production rates are equal, under the steady-state assumption, the flux-sum serves as a proxy of the metabolite pool size in the system, by which the cellular ATP/NADPH ratio could be examined. The analysis revealed a narrow range of intracellular ATP pool (Table 1). In contrast, a larger variation in NADPH levels was determined. We computed that biomass accumulation required an ATP/NADPH ratio of 2.11 and 19.49, under autotrophic and mixotrophic conditions, respectively. In comparison, all the examined alkenes required ATP/NADPH ratios below the values for biomass (Table 1). Therefore, in order to attain a growth-coupled production, genetic interventions are needed to manipulate the cofactor pools^41^. A lower ATP availability could be achieved by introducing an “ATP-wasting” mechanism (e.g., ATPM)^42^. A higher cofactor availability of NAD(P)H could be achieved by the introduction of non-native NADH-dependent oxidoreductases, or protein engineering of enzymes for swapped cofactor-dependency^43^.

It should be noted that, the ATP/NADPH ratios presented here slightly differ from earlier published results^44,45^. These differences could be justified by that: (i) Here, we considered flux in the entire metabolic network, whereas previous studies mainly considered photosynthetic reactions (e.g., ATPSu and NDH-1 and FNOR). (ii) Different metabolic network sized were examined, covering different number of reactions acting on ATP and NAD(P)H: 760 reactions^44^, 780 reactions^45^, and 883 reactions in this study. (iii) We simulated a synergistic interaction between biomass formation (10%) and alkene biosynthesis, whereas earlier studies modelled 0%^44^ or 75%^45^ of growth, which affected the evaluation of ATP/NAD(P)H demand.

### Evaluation of intracellular flux distributions

In order to identify the metabolic reactions contributing to the computed turnover rates, we employed a reaction-centric approach by using pFBA. By comparing biomass and alkene-overproducing strains, potential reactions whose metabolic fluxes constrain alkenes synthesis in *Synechocystis* could be recognized. We would like to note that FBA and ^13^C-metabolic flux analysis (MFA) are two different approaches to determine the intracellular fluxes^46^. Further, our model does not include information regarding cellular regulation or enzyme kinetics, which influence the conclusion on the flow of carbon in the cell.

#### Cofactor energy metabolism

Under both trophic growth modes, the majority of ATP generation was performed by the photosynthetic ATP synthetase (ATPSu) (Table 3). Under autotrophic conditions, a residual respiratory activity (ATPS4rpp_1) was determined for all objectives, but for the 1-undecene production. Our data are in line with earlier studies, showing that the respiratory pathway in *Synechocystis* is active also during light, albeit at low capacity^47^. Isoprene and ethylene production required a higher contribution of respiratory oxidative phosphorylation, whereas for isobutene, pyruvate kinase (PYK), from lower glycolysis, contributed to ATP generation (Table 3).

**Table 3:** Predicted flux distributions of cofactors-producing reactions.

		Biomass		Isoprene		Isobutene		Ethylene		1-undecene	
Trophy	Reaction	Flux	Percent	Flux	Percent	Flux	Percent	Flux	Percent	Flux	Percent
ATP											
Auto	ATPSu	16.04	98.28	16.19	96.85	15.84	91.98	15.04	98.09	14.46	99.95
	PYK	0.15	0.91	0.01	0.08	1.37	7.95	0.00	0.00	0.01	0.04
	ATPS4rpp_1	0.13	0.80	0.51	3.07	0.01	0.07	0.29	1.90	0.00	0.00
Mixo	ATPSu	10.70	99.99	10.50	100.00	11.10	95.25	13.44	98.65	10.76	99.96
	PYK	0.00	0.00	0.00	0.00	0.55	4.75	0.00	0.00	0.00	0.04
	ATPS4rpp_1	0.00	0.00	0.00	0.00	0.00	0.00	0.18	1.35	0.00	0.00
NADPH											
Auto	FNOR	7.56	97.66	10.15	99.82	10.92	99.83	7.50	88.09	10.94	99.77
	ICDHyr	0.10	1.33	0.01	0.10	0.01	0.09	0.98	11.56	0.01	0.09
	ME2	0.00	0.00	0.00	0.00	0.00	0.00	0.02	0.27	0.01	0.06
	MTHFD	0.06	0.78	0.01	0.06	0.01	0.06	0.01	0.07	0.01	0.05
Mixo	FNOR	0.02	3.02	1.76	94.84	3.20	86.62	3.73	69.72	2.37	99.28
	ICDHyr	0.07	12.76	0.01	0.38	0.01	0.19	1.24	23.14	0.01	0.29
	ME2	0.05	8.67	0.00	0.26	0.48	13.06	0.38	7.05	0.00	0.20
	MTHFD	0.04	7.49	0.00	0.22	0.00	0.09	0.00	0.08	0.00	0.17
	G6PDH2r	0.23	41.13	0.08	4.24	0.00	0.00	0.00	0.00	0.00	0.00
	GND	0.14	24.69	0.00	0.00	0.00	0.00	0.00	0.00	0.00	0.00
NADH											
Auto	GLYCLc	9E−03	4E + 00	9E−04	4E + 00	0E + 00	0E + 00	0E + 00	0E + 00	0E + 00	0E + 00
	HISTD	1E−02	6E + 00	1E−03	6E + 00	1E−03	2E−01	1E−03	1E−01	1E−03	8E + 00
	IMPD	1E−02	5E + 00	1E−03	5E + 00	1E−03	2E−01	1E−03	1E−01	1E−03	7E + 00
	IPMD	4E−02	2E + 01	4E−03	2E + 01	7E−01	1E + 02	4E−03	4E−01	4E−03	2E + 01
	MDH	7E−02	3E + 01	7E−03	3E + 01	7E−03	1E + 00	6E−01	7E + 01	0E + 00	0E + 00
	PGCD	1E−01	4E + 01	1E−02	4E + 01	1E−02	2E + 00	1E−02	1E + 00	1E−02	6E + 01
	P5CD	0E + 00	0E + 00	0E + 00	0E + 00	0E + 00	0E + 00	3E−01	3E + 01	0E + 00	0E + 00
Mixo	GLYCLc	6E−03	4E + 00	6E−04	5E + 00	0E + 00	0E + 00	6E−04	6E−02	0E + 00	0E + 00
	HISTD	1E−02	7E + 00	1E−03	8E + 00	1E−03	2E−01	1E−03	9E−02	1E−03	8E + 00
	IMPD	9E−03	7E + 00	9E−04	7E + 00	9E−04	2E−01	9E−04	8E−02	9E−04	7E + 00
	IPMD	3E−02	2E + 01	3E−03	2E + 01	5E−01	1E + 02	3E−03	3E−01	3E−03	2E + 01
	MDH	0E + 00	0E + 00	0E + 00	0E + 00	0E + 00	0E + 00	4E−01	4E + 01	0E + 00	0E + 00
	PGCD	8E−02	6E + 01	7E−03	5E + 01	6E−03	1E + 00	2E−01	2E + 01	8E−03	6E + 01
	P5CD	0E + 00	0E + 00	0E + 00	0E + 00	0E + 00	0E + 00	4E−01	4E + 01	0E + 00	0E + 00

Presented are percentage (%) and the corresponding flux (mmol/gDW/h) of reactions contributing to the generation of ATP and NAD(P)H. Zero flux indicates that the reaction carries no flux or <0.01 mmol/gDW/h. For the complete list of reactions, including their abbreviations, subsystem, lower and upper bounds, and stoichiometry, refer to data availability section.

*Auto* autotrophic, *Mixo* mixotrophic.

Reactions involved in ATP consumption were more diverse than for ATP generation and differed between growth conditions. Under autotrophic growth, ATP consumption was dictated by reactions within CBB cycle and glycolysis/gluconeogenesis (phosphoglycerate kinase, PGK and phosphoribulokinase, PRUK) for all strains (Table 4). Earlier in vivo studies also determined PGK and PRUK as major ATP-consumers in *Synechocystis*^48,49^. Our data also revealed specific flux distributions depending on the objective function. For ethylene production, the ATP-consuming reactions were within nitrogen metabolism pathways (e.g., glutamine synthetase, GLNS), whereas for isoprene production, the terpenoid biosynthesis pathways contributed the most to ATP consumption. Bicarbonate transport (BCT1_syn) exhibited a large ATP consumption for production of isoprene and 1-undecene. Acetyl coenzyme A (acetyl-CoA) carboxylase (ACCOAC) was mainly involved in 1-undecene accumulation, probably due to high amount of carbon assimilated during the elongation process of fatty acyl chain for 1-undecene synthesis.

**Table 4:** Predicted flux distributions of cofactors-consuming reactions.

		Biomass		Isoprene		Isobutene		Ethylene		1-undecene	
Trophy	Reaction	Flux	Percent	Flux	Percent	Flux	Percent	Flux	Percent	Flux	Percent
ATP											
Auto	PGK	−6.37	39.05	−8.02	47.97	−10.12	58.80	−4.89	35.87	−7.84	54.15
	PRUK	−3.50	21.43	−4.38	26.18	−5.77	33.52	−2.88	21.11	−3.95	27.29
	GLNS	−0.11	0.68	−0.01	0.07	−0.01	0.06	−1.09	8.00	−0.02	0.10
	ARGSS	−0.02	0.14	0.00	0.01	0.00	0.01	−0.41	3.02	0.00	0.02
	ACGK	−0.02	0.14	0.00	0.01	0.00	0.01	−0.41	3.02	0.00	0.02
	ACCOAC	−0.24	1.49	−0.02	0.15	−0.02	0.14	−0.02	0.12	−1.52	10.53
	BCT1_syn	0.00	0.00	−2.34	13.98	0.00	0.00	0.00	0.00	−0.54	3.72
	ADK1	−0.17	1.06	−0.02	0.10	−0.02	0.10	−1.01	7.38	−0.02	0.12
Mixo	PRUK	-0.17	1.56	−0.41	3.94	−2.06	17.63	−2.88	21.11	−0.19	1.76
	PGK	0.00	0.00	−0.45	4.31	−3.04	26.10	−4.89	35.87	−0.34	3.13
	GLNS	−0.52	4.85	−0.05	0.47	−0.05	0.43	−1.09	8.00	−0.05	0.48
	ARGSS	−0.02	0.14	0.00	0.01	0.00	0.01	−0.41	3.02	0.00	0.01
	ACGK	−0.02	0.14	0.00	0.01	0.00	0.01	−0.41	3.02	0.00	0.01
	ACCOAC	−0.17	1.55	−0.02	0.16	−0.02	0.14	−0.02	0.12	−0.95	8.82
	ADK1	−0.12	1.10	−0.01	0.11	−0.01	0.10	−1.01	7.38	−0.01	0.11
	CYPHYS	−6.08	56.83	0.00	0.00	−5.27	45.18	0.00	0.00	−8.53	79.18
	ATPM	0.00	0.00	−7.96	75.80	0.00	0.00	0.00	0.00	0.00	0.00
NADPH											
Auto	GAPDi_nadp	−6.37	82.30	−8.02	78.86	−10.12	92.53	−7.78	91.35	−7.84	71.43
	KARA1	−0.07	0.89	−0.01	0.07	−0.68	6.26	−0.01	0.08	−0.01	0.06
	3OAR60	−0.03	0.44	0.00	0.03	0.00	0.03	0.00	0.04	−0.30	2.77
	DXPRIi	−0.01	0.19	−0.67	6.61	0.00	0.01	0.00	0.02	0.00	0.01
Mixo	GAPDi_nadp	0.00	0.00	−0.45	24.46	−3.04	82.44	−4.89	91.33	−0.34	14.13
	KARA1	−0.05	8.55	0.00	0.25	−0.52	14.03	0.00	0.09	0.00	0.20
	3OAR60	−0.02	4.25	0.00	0.13	0.00	0.06	0.00	0.04	−0.19	7.92
	DXPRIi	−0.01	1.83	−0.41	22.22	0.00	0.03	0.00	0.02	0.00	0.04
NADH											
Auto	NDH1_2u	−0.11	44.84	−0.01	44.84	−0.69	97.87	−0.59	60.30	0.00	0.00
	HSDxi	−0.05	21.52	−0.01	21.52	−0.01	0.75	−0.01	0.54	−0.01	30.12
	ALAD_L	−0.04	15.73	0.00	15.73	0.00	0.00	0.00	0.21	0.00	11.73
	P5CRx	−0.02	7.95	0.00	7.95	0.00	0.28	0.00	0.20	0.00	11.13
	GLUSx	0.00	0.00	0.00	0.00	0.00	0.00	−0.37	38.31	0.00	22.77
	MTHFR2	−0.01	4.13	0.00	4.13	0.00	0.14	0.00	0.10	0.00	5.78
Mixo	HSDxi	−0.04	26.42	0.00	29.81	0.00	0.68	0.00	0.33	0.00	28.43
	ALAD_L	−0.03	19.28	0.00	21.76	0.00	0.38	0.00	0.15	0.00	0.00
	P5CRx	−0.01	9.76	0.00	11.01	0.00	0.25	0.00	0.12	0.00	10.50
	GLUSx	−0.04	32.29	0.00	23.56	−0.04	7.34	−1.08	99.07	0.00	31.96
	MTHFR2	−0.01	5.07	0.00	5.72	0.00	0.13	0.00	0.06	0.00	5.46

Presented are percentage (%) and the corresponding flux (mmol/gDW/h) of reactions contributing to the consumption of ATP and NAD(P)H. Zero flux indicates that the reaction carries no flux or <0.01 mmol/gDW/h. For the complete list of reactions, including their abbreviations, subsystem, lower and upper bounds, and stoichiometry, refer to data availability section.

*Auto* autotrophic, *Mixo* mixotrophic.

Reactions involved in ATP consumption were more diverse than for ATP generation and differed between growth conditions. Under autotrophic growth, ATP consumption was dictated by reactions within CBB cycle and glycolysis/gluconeogenesis (phosphoglycerate kinase, PGK and phosphoribulokinase, PRUK) for all strains (Table 4). Earlier in vivo studies also determined PGK and PRUK as major ATP-consumers in *Synechocystis*^48,49^. Our data also revealed specific flux distributions depending on the objective function. For ethylene production, the ATP-consuming reactions were within nitrogen metabolism pathways (e.g., glutamine synthetase, GLNS), whereas for isoprene production, the terpenoid biosynthesis pathways contributed the most to ATP consumption. Bicarbonate transport (BCT1_syn) exhibited a large ATP consumption for production of isoprene and 1-undecene. Acetyl coenzyme A (acetyl-CoA) carboxylase (ACCOAC) was mainly involved in 1-undecene accumulation, probably due to high amount of carbon assimilated during the elongation process of fatty-acyl chain for 1-undecene synthesis.

Under mixotrophic conditions, the ATP consumption was mainly governed by cyanophycin metabolism (cyanophycin synthetase, CYPHYS) during biomass, isobutene and 1-undecene synthesis (Table 4). For isoprene accumulation, ATP maintenance (ATPM) accounted for the majority of ATP consumption (Table 4). As ATPM contributes to cellular functions other than growth (e.g., turgor pressure) (Fig. 3b), it can be inferred that a hyper-producing isoprene strain would more likely become ATP-limited when grown mixotrophically, as compared to the other alkene-producing strains.

**Fig. 4:**
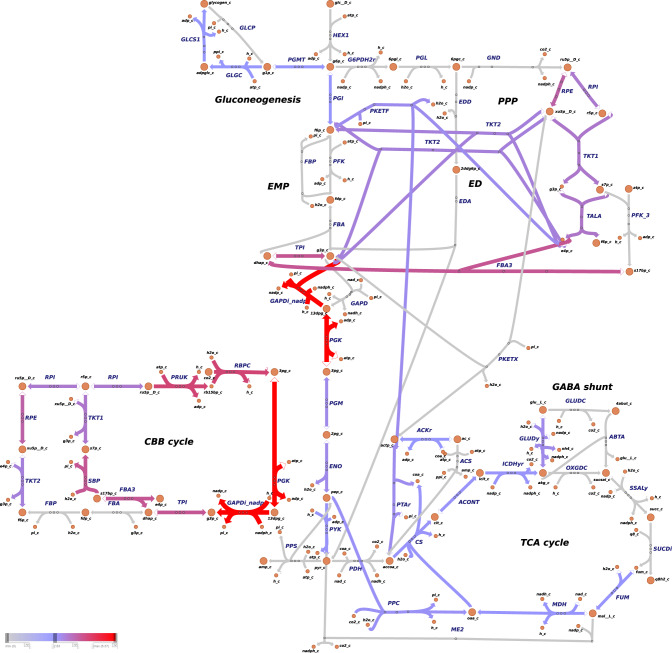
Metabolic flux map of central carbon metabolism for *Synechocystis* sp. PCC 6803. *Synechocystis* was simulated to grow under autotrophic conditions, with biomass set as objective. Reaction rates (mmol/gDW/h) were predicted using pFBA^69^. Note that, the colors associated with the fluxes are relative to the other reactions rates presented in the map. Irreversible reactions are indicated by one-headed arrows; reversible reactions are indicated by two-headed arrows. For reaction directionality, refer to the data availability section. For metabolic flux maps of central carbon metabolism for biomass simulated to grow mixotrophic conditions, and other objectives simulated to grow under auto- and mixotrophic conditions, refer to Supplementary Figs. 1–9. The map was generated with Escher web-tool^72^. Metabolic reactions and metabolites are indicated by their BiGG identifier^73^. TCA tricarboxylic acid, GABA branched-chain amino acids γ-aminobutyrate, CBB Calvin–Benson–Bassham, ED Entner–Doudoroff, EMP Embden-Meyerhof-Parnas, PPP pentose phosphate pathway.

**Fig. 5:**
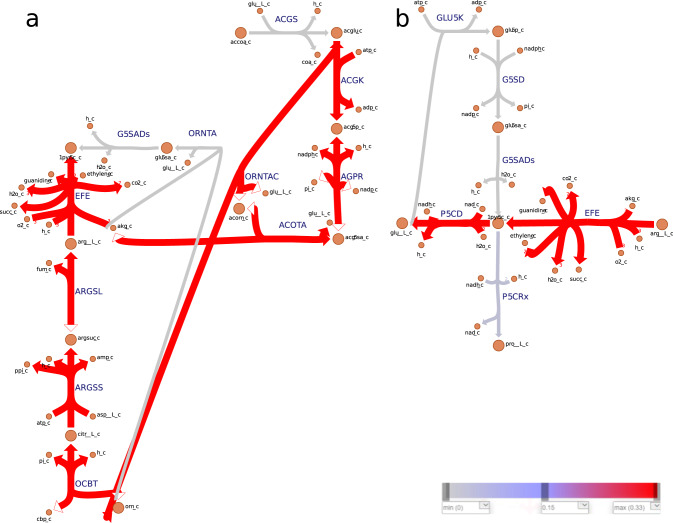
Metabolic flux map of nitrogen metabolism for *Synechocystis* sp. PCC 6803. *Synechocystis* was simulated to grow under autotrophic conditions, with ethylene set as objective. Reaction rates (mmol/gDW/h) were predicted using pFBA^69^. **a** L-glutamate and L-arginine regeneration through the urea cycle. **b** L-glutamate and L-proline regeneration. Note that, the colors associated with the fluxes are relative to the other reactions rates presented in the map. Irreversible reactions are indicated by one-headed arrows; reversible reactions are indicated by two-headed arrows. For reaction directionality see data availability section of the GitHub repository. For metabolic flux maps of nitrogen metabolism for *Synechocystis* sp. PCC 6803 with ethylene set as objective, simulated to grow under mixotrophic conditions, refer to Supplementary Fig. 10. The map was generated with Escher web-tool^72^. Metabolic reactions and metabolites are indicated by their BiGG identifier^73^.

**Fig. 6:**
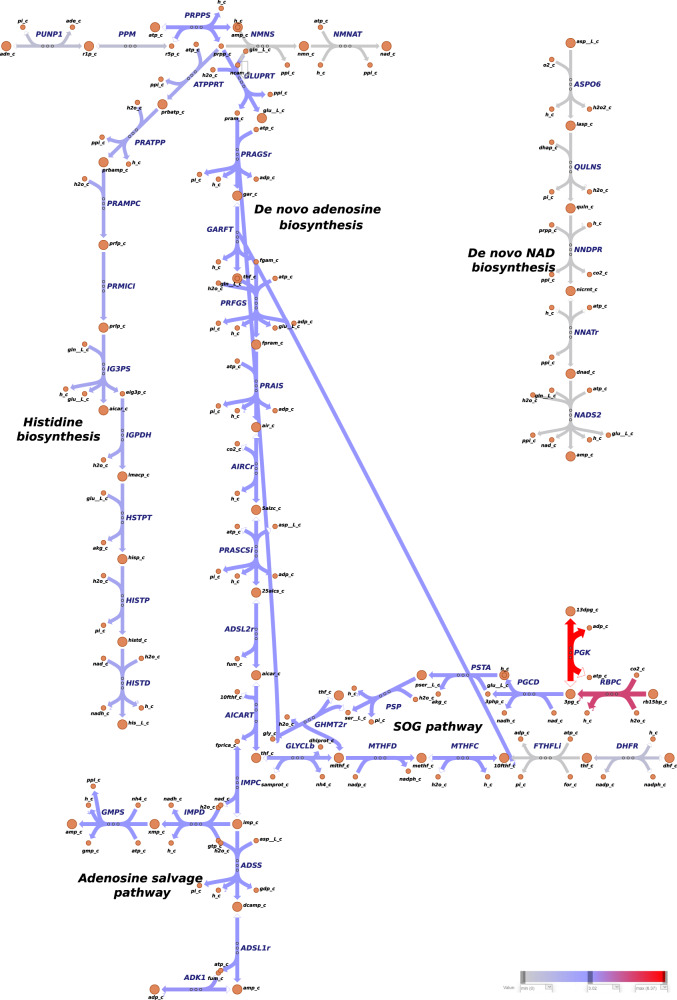
Metabolic flux map of ATP and NAD de novo and salvage metabolism for *Synechocystis* sp. PCC 6803. *Synechocystis* was simulated to grow under autotrophic conditions, with biomass set as objective. Reaction rate (mmol/gDW/h) were predicted using pFBA^69^. Note that, the colors associated with the fluxes are relative to the other reactions rates presented in the map. Irreversible reactions are indicated by one-headed arrows; reversible reactions are indicated by two-headed arrows. For reaction directionality, refer to the data availability section. For metabolic flux maps of central carbon metabolism for biomass simulated to grow mixotrophic conditions, and other objectives simulated to grow under auto- and mixotrophic conditions, refer to Supplementary Figs. 11–19. The map was generated with Escher web-tool^72^. Metabolic reactions and metabolites are indicated by their BiGG identifier^73^. NAD nicotinamide adenine dinucleotide, SOG serine, one-carbon cycle, glycine synthesis.

We additionally investigated how the ATP precursors (ADP, AMP and Pi) are recycled within the metabolic network (Fig. 6, Supplementary Tables 9–12 and Supplementary Figs. 11–19). The stoichiometric analysis revealed that, under both autotrophic and mixotrophic growth, the de novo adenosine biosynthesis, downstream to phosphoribosylpyrophosphate synthetase (PRPPS), contributed equally to all objectives but ethylene production (Fig. 6, Supplementary Table 9 and Supplementary Figs. 11–19). The salvage adenosine pathway through adenylate kinase (ADK1) contributes the most to ADP-AMP cycling during ethylene (Supplementary Tables 7 and 10 and Supplementary Figs. 16–17) and biomass overproduction (Fig. 6 and Supplementary Table 7). For ethylene synthesis, argininosuccinate synthase (ARGSS) in arginine metabolism showed enhanced flux to produce AMP (Fig. 5a, Supplementary Fig. 10 and Supplementary Table 9). Of note, phosphoketolase (PKETF) was shown to be a common consumer of Pi for the production of all objective functions (Fig. 4, and Supplementary Table 12).

The enhanced ADK1 flux during biomass (Fig. 6, Supplementary Fig. 11 and Supplementary Tables 7 and 10) and ethylene production (Supplementary Figs. 16–17 and Supplementary Tables 7 and 10) presumably sustains the increase in respiration rate, by providing more ADP to the oxidative phosphorylation pathway. A prior study revealed that low respiration rate is a possible limiting factor of ethylene production through ethylene-forming enzyme (EFE) in *Saccharomyces cerevisiae*^50^, and could explain the low ethylene production found experimentally in *Synechocystis*^36^.

Based on our studies and others^51^, we suggest that a combinatorial metabolic engineering, with the aim to form an efficient ATP cycling for fueling the CBB cycle, could be based on the ATP-consuming enzymes (e.g., PRUK and PGK), and on the ATP-producing enzymes (i.e., PYK). In addition a high ATP turnover would be beneficial for the heterologous production of alkenes, and probably other products, in *Synechocystis*, as previously confirmed for *E. coli*^42^.

We propose that, under mixotrophic conditions, the regeneration of cellular nitrogenous polymers (e.g., cyanophycin) is essential for optimized nitrogen and energy metabolism in the cell. The data are in agreement with previous studies, arguing that the transition in growth conditions affects nitrogen metabolism through cyanophycin^52^.

#### Cofactor redox metabolism

In agreement with earlier reports^10,53^, the flux analysis revealed that, under both autotrophic and mixotrophic growth, the photosynthetic ferredoxin-NADP^+^ reductase (FNOR) contributed largely to the supply of NADPH (Table 3). An exception was noted for biomass production, under mixotrophic conditions, for which NADPH was produced by the oxidative pentose phosphate pathway (Table 3). In relation to the calculated ATP/NADPH ratios (Table 1), we presume that the high ratio determined for biomass production under mixotrophic conditions stems from the lower flux through the FNOR, as compared to that as seen for the other objectives.

In the ethylene-overproducing strain, the flux through isocitrate dehydrogenase (ICDHyr) was significantly higher than that in the other modeled strains (Table 3 and Supplementary Figs. 6–7). We suggest that ICDHyr is a good candidate for enhanced production of TCA-derived molecules, such as ethylene, supported by the results from studies on engineered ethylene-producing strain of *Synechocystis*^54^.

The flux distribution of reactions catalyzing NADPH consumption revealed that for all objectives, NADPH was mainly consumed by glyceraldehyde-3-phosphate dehydrogenase (GAPDi_nadp), a key enzyme within the CBB cycle and gluconeogenesis (Table 4). As could be expected, the flux though ketol-acid reductoisomerase (KARA1) within the branched-chain amino acids (BCAA) biosynthesis was enhanced for isobutene production, and reaction fluxes though the terpenoid and fatty acids pathways (i.e., fatty-acyl reductases) were increased for isoprene and 1-undecene.

Though carrying low flux under both growth conditions, production of NADH were distributed among BCAA biosynthesis pathway (3-isopropylmalate dehydrogenase, IPMD), TCA cycle (malate dehydrogenase, MDH) and L-arginine and L-proline metabolism (phosphoglycerate dehydrogenase, PGCD) (Table 3). Notably, an enzyme from the L-arginine and L-proline metabolism (Δ1-pyrroline-5-carboxylate dehydrogenase, P5CD) was found to be predominant during ethylene overproduction (Fig. 5b).

We observed that under autotrophy, for all objectives but 1-undecene, the photosynthesis (NAD(P)H dehydrogenase, NDH1_2u) is the main NADH consumer (Table 4). For 1-undecene, L-lysine metabolism (L-homoserine dehydrogenase, HSDxi) contributed the most. HSDxi and L-alanine metabolism (L-alanine dehydrogenase, ALAD_L) were major NADH consumers for biomass, isoprene, isobutene and 1-undecene production. For ethylene production, nitrogen metabolism was a key NADH consumer (e.g., L-glutamate synthase, GLUSx).

The data imply that the regeneration of the NADH pool in the system is facilitated mainly by amino acids metabolism. The reduction of nitrate to ammonia requires a considerable amount of NADPH. We suggest that designing an alternative NADH-dependent pathway for ammonium assimilation (for example by introducing ALAD_L) could reduce the competition for NADPH. Hence, increasing the potential for NADPH-dependent product formation (Fig. 1), while simultaneously enabling nitrogen cycling in the cell.

We additionally investigated the redox balancing mechanism by the (de)phosphorylating enzymes, and identified that NAD kinase (NADK) had a negligible contribution to the overall flux of NAD^+^ consumption (Supplementary Table 7). As NADP^+^ serves as the final electron acceptor of photosystem I^10^, this finding was rather surprising. The role of NADK only begins to be elucidated^55,56^, and the functionality of NADP^+^ phosphatase remains to be discovered.

Considering the large variation in NADPH turnover rates (Table 1), we postulate that, under light-limited conditions, the ATP/NADPH ratios are mainly governed by the reduction of NADP^+^, rather than by the regeneration of ATP. Our data are consistent with a previous study, demonstrating that, under steady-state photosynthesis, CO_2_ assimilation is not limited by the availability of ATP, but by the rate of NADPH formation^57^.

#### Energy and redox balance are interlinked with the production of central metabolites

Our findings show that balancing the intracellular ratio of ATP and NADPH is directly interconnected with and influence the regeneration of central carbon metabolites (i.e., pyruvate and acetyl-CoA). We identified that, under autotrophic conditions, carbon is channeled back to the Embden-Meyerhof-Parnas (EMP) pathway to synthetize glycogen through glucose-1-phosphate adenylyltransferase (GLGC) and glycogen synthase (GLCS1) reactions (Fig. 4 and Supplementary Figs. 2, 4, 6). In *Synechocystis*, glycogen metabolism was shown to act as an ATP-buffering system, and the inactivation of GLGC led to an increased NADPH pool^58,59^. It has furthermore been reported that glycolytic routes form anaplerotic shunts that replenish metabolites for carbon fixation by the CBB cycle^60^. We suggest that gluconeogenesis and glycogen turnover should be considered within future bioengineering research. This is in contrast to a number metabolic engineering studies, in which a glycogen-deficient *Synechocystis* strain is used as chassis for production^61,62^.

Pyruvate kinase (PYK) and Malic enzyme (ME2) are the major contributors to pyruvate synthesis, although at distinct ratios, depending on the objective function (Supplementary Table 13). The data are in accordance with previous reports, suggesting that due to allosteric-inhibition of PYK by its product, ATP^63^, ME2 functions as an additional route for synthesizing pyruvate in *Synechocystis*^7^. Fluxomic studies pinpointed that PYK reaction represents a bottleneck for isobutyraldehyde production in *Synechococcus elongatus* PCC 7942, which was overcome by heterologous overproduction of PYK^64^. Under mixotrophic conditions, the Entner–Doudoroff pathway also provided pyruvate for biomass synthesis (Supplementary Table 13). Hence, based on pure stoichiometry, and in agreement with previous research, we suggest that PYK and ME2 are good candidates for metabolic engineering of *Synechocystis* for hydrocarbon production. The CO_2_ released by ME2 can be further assimilated by phosphoenolpyruvate carboxylase (PPC) (Fig. 4), thus resulting in a net zero carbon loss and a high product yield.

Interestingly, and less intuitively, IPMD within the isobutene biosynthesis pathway (Fig. 1), showed a high contribution for redox recycling in biomass, isoprene and 1-undecene biosynthesis as well (Table 3). In cancer research, it was shown that the TCA cycle in tumor cells is heavily fed by acetyl-CoA derived from BCAA^65^. Taken together, we presume that, the catabolism of BCAA provides an alternative route for the synthesis of acetyl-CoA - a key precursor molecule for many cellular biosynthesis pathways, including terpenoids and lipids.

Our results further emphasize the essentiality of metabolic shortcuts in central carbon metabolism for attaining effective metabolic states^66^. We show that acetate metabolism through phosphoketolase serves as an efficient carbon- and energy-recycling pathway for cell maintenance, as suggested in a biochemical study focusing on the role of phosphoketolase in cyanobacteria^67^.

#### Serine and glycine metabolism represents an uncharacterized pathway for cellular energy and redox homeostasis in Synechocystis

We show that FBA is a powerful tool to investigate cellular metabolism globally and highlight flux patterns, by which new routes could be found and serve as basis for future studies. We learned that reactions participating in the L-serine synthesis, one-carbon metabolism and the glycine cleavage system (SOG pathway) function as an auxiliary pathway to regenerate ATP, NADPH and NADH in *Synechocystis*. Fluxes through SOG were identified when maximizing biomass and alkenes production (Fig. 6, Supplementary Tables 9–12 and Supplementary Figs. 11–19). The discovery that SOG pathway contributes to the biosynthetic requirements of ATP and NADPH of high-proliferating cells^68^, strengthens our suggestion that SOG deserves a thorough investigation for metabolic engineering of *Synechocystis*.

To conclude, we demonstrate the significance of high flexibility in activity of energy- and carbon-converting enzymes in order to achieve the required ATP/NADPH output ratio. It was apparent that the overall activity of the *Synechocystis* metabolism is governed by several high-flux core reactions, which probably allows the cell to adapt rapidly to perturbations in environmental conditions and genetic modifications. We also show that assessment of ATP and NAD(P)H balance can neither be done in isolation from each other and their precursors, nor from carbon and nitrogen metabolism. Even though the predictions are yet to be validated in vivo, our results provide hints for strain design strategies enabling efficient bioproduction of valuable compounds derived from core metabolic pathways in *Synechocystis*.

## Methods

### Computational tools

Parsimonious enzyme usage FBA (pFBA)^69^ was employed on COBRApy v0.20.0^70^ using the commercial solver Gurobi v10.0.1 (Gurobi Optimization, Inc., Houston, TX, United States) in Python v3.8.5, using the packages Pandas v1.3.0, NumPy v1.19.4, Matplotlib v3.3.3 and Pickle v4.0. Model consistency was tested with MEMOTE v0.12.0^71^. Visualization of the metabolic reaction network and the obtained fluxes was done using the Escher web-tool^72^.

### Metabolic network reconstruction

The iJN678 genome-scale metabolic model of *Synechocystis* sp. PCC 6803^19^, downloaded from the BiGG database^73^, was used for the computational analysis. The model reconstruction includes 622 genes, 863 reactions and 795 metabolites. iJN678 also contains three different biomass compositions, allowing for simulating autotrophic, mixotrophic, and heterotrophic growth conditions, in addition to constraining the appropriate carbon and photon uptake rates.

The metabolic network was updated in accordance with the literature, yielding iJN678_AK, (Supplementary Table 1), as follows: addition of the tricarboxylic acid (TCA) cycle shunt reactions^74–76^, addition of the phosphoketolase reactions^67,77^, addition of the Entner–Doudoroff (ED) pathway^78^, addition of the light-independent L-serine biosynthesis pathway^79^, addition of prephenate transaminase, prephenate dehydratase and arogenate dehydrogenase^80^, transhydrogenase (NADTRHD) was changed to a reversible reaction^11^, addition of NADH dehydrogenase 2, alternative respiratory terminal oxidase, and flavodiiron proteins 2 and 4, and constraining cytochrome b6f, cytochrome c oxidase and NAD(P)H dehydrogenase to zero^47,81^. In addition, non-growth associated ATP maintenance (ATPM)^82,83^ reaction was added to the model. The ATPM coefficient is represented by a pseudo-reaction that degrades ATP into ADP and orthophosphate (Pi) (Eq. 1).

1 $${\rm{ATP}}+{{\rm{H}}}_{2}{\rm{O}}\to {\rm{ADP}}+{\rm{Pi}}+{\rm{H}}$$

For the evaluation of heterologous alkene production in *Synechocystis*, four biosynthesis pathways^34–36,84^ were separately implemented into the iJN678_AK model, yielding iJN678_AK_isoprene, iJN678_AK_isobutene, iJN678_AK_ethylene and iJN678_AK_1-undecene (Supplementary Tables 2–5). In each reconstructed model, a cytoplasmatic export reaction for each alkene was introduced and set as the objective function. The resulting metabolic network reconstructions are encoded in SBML format (Systems Biology Markup Language)^85^, and are available in the GitHub repository https://github.com/amitkugler/CBA.

### Constraint-based modeling

To evaluate the steady-state flux distributions, we employed pFBA^69^, in which the objective function is optimized using FBA, followed by the minimization of total absolute flux through all gene-associated reactions (Eqs. 2–5).

2 $${maximize}\,Z={c}^{T}\,\cdot\, v$$

s.t

3 $$\mathop{\sum }\limits_{j=1}^{n}{S}_{{ij}}\,\cdot\, {v}_{j}=0$$

4 $$\,{{v}_{j}}^{{LB}}\le {v}_{j}\le {{v}_{j}}^{{UB}}$$

s.t

5 $${minimize}\mathop{\sum }\limits_{j=1}^{m}\left|{v}_{{irrev},\,j}\right|$$

Where $Z$ is the objective function, $v$ is the flux vector of the metabolic reactions, ${c}^{T}$ is the transposed vector of the objective coefficient. ${S}_{{ij}}$ refers to the stoichiometric coefficient of metabolite i participating in reaction j, and the ${v}_{j}$ refers to the vector of reaction flux (mmol/gDW/h; gDW, gram dry weight) of reaction j at steady state. The flux ${v}_{j}$ is the j-th component of an n-dimensional flux vector v, where n is the total number of fluxes, m is the number of gene-associated irreversible reactions (${v}_{{irrev}}$). LB and UB correspond to the lower bound and upper bound, respectively, of the j-th reaction in the flux vector *v*. Equation 3 can also be used to distinguish between reversible and irreversible reactions, where ${{v}_{j}}^{{LB}}=0$ for the latter.

To explore the flux solution space, flux variability analysis (FVA)^86^ was performed. The fraction of the optimum was set to 95%, which accounts for 5% variation around the best-known objective value. The FVA revealed infinite bounds for transhydrogenase (NADTRHD), irreversible leucine transaminase (LEUTAi) and glycine cleavage system (GLYCL and GLYCL_2). It was reported earlier that, the transhydrogenase reaction was poorly resolved by ^13^C measurements^87^. Therefore, we chose to model redox balance without an active NADTRHD, as previously suggested^88^. At the same time, we could learn how the entire metabolic network balances between NADPH/NADH. In addition, the upper and lower bounds of LEUTAi and GLYCL and GLYCL_2 were constrained to zero, and the glycine cleavage system was modeled by introducing three separate reactions (Supplementary Table 1).

In order to avoid thermodynamic infeasible cycles, the “add_loopless” function in COBRApy was applied, that makes thermodynamically infeasible loops impossible^89^. In addition, we coupled our analysis to FVA.

According to previous studies^7,90^, autotrophic metabolism was simulated by constraining the carbon dioxide, bicarbonate and glucose uptake rates to 0, 3.7 and 0 mmol/gDW/h, respectively. Mixotrophic metabolism was simulated by constraining the inorganic carbon (carbon dioxide and bicarbonate) uptake rates to 0 and glucose uptake rate to 0.38, respectively. For both trophic conditions, the photon uptake rate to was set to 45 mmol/gDW/h, to simulate a light-limited condition. These settings resulted in maximum specific growth rates of 0.082 h^−1^ and 0.056 h^−1^, under autotrophic and mixotrophic conditions, respectively, which are in agreement with experimental growth values^7,22^. In addition, the biomass objective function^91^ was set to BIOMASS_Ec_SynAuto, BIOMASS_Ec_SynMixo, depending on the simulated growth conditions, to account for changes in macromolecular composition.

### Two-step optimization strategy

Following earlier studies^92,93^, showing that a feasible overproduction of hydrocarbons in *Synechocystis* entails a carbon partitioning to biomass of 10%, we simulated a scenario where the cellular growth is limited, while achieving a high alkene production rate. We used a two-step optimization strategy, where, first, the biomass objective function was maximized. Then, the minimum flux through the biomass objective function was set to 10% of the obtained value, followed by the maximization of alkene production.

### Evaluation of production strains

For providing a global perspective on the relation between growth rate and alkene production a phenotypic phase plane analysis^38^ was carried out.

The flux-sum analysis was used to quantify the cofactors turnover rates among the metabolic network^39^. The flux-sum values for a metabolite represent the sum of flux in all the reactions that consume or produce it, multiplied by the corresponding stoichiometric coefficients (Eq. 6).

6 $$\,{\varPhi }_{i}=\sum _{j\varepsilon {P}_{i}}{S}_{{ij}}\bullet {v}_{j}=\sum _{j\varepsilon {C}_{i}}{S}_{{ij}}{\bullet v}_{j}=0.5\sum _{j}{\rm{|}}{S}_{{ij}}\bullet {v}_{j}{\rm{|}}$$

Where ${P}_{i}\,$ denotes the set of reactions producing metabolite i, and ${C}_{i}$ denotes the set of reactions consuming metabolite i. Under the steady-state conditions, the consumption and production rates for any metabolite are equal. Thus, the turnover rate of metabolite i is half the absolute sum of consumption and the generation rates.

The theoretical maximum productivity is expressed h^−1^ or mmol/gDW/h for biomass and alkenes, respectively. The theoretical maximum yield is expressed as gram product produced per gram of substrate consumed. The carbon-conversion efficiency was estimated by calculating carbon-mole (c-mole) produced per c-mole of substrate consumed.

### Reporting summary

Further information on research design is available in the Nature Research Reporting Summary linked to this article.

### Supplementary information

Supplementary material

Reporting Summary

## Data Availability

All the data supporting the work are available within the paper, its supplementary material, and in the GitHub repository https://github.com/amitkugler/CBA.

## Code availability

The models and the code used for this study can be found in the GitHub repository https://github.com/amitkugler/CBA.
