# Supplementary material for: Optimal energy and redox metabolism in the cyanobacterium Synechocystis sp. PCC 6803
Source: NPJ Syst Biol Appl. 2023 Sep 22;9:47. doi: 10.1038/s41540-023-00307-3 (PMC10516873; doi:10.1038/s41540-023-00307-3)
Supplement: Supplementary file 1 — Supplementary material [file 41540_2023_307_MOESM1_ESM.pdf]

Supplementary material (figures) for Optimal energy and redox metabolism in the cyanobacterium *Synechocystis* sp. PCC 6803

Amit Kugler[1], Karin Stensjö[1]*

[1]Microbial Chemistry, Department of Chemistry-Ångström Laboratory, Uppsala University, Box 523, SE-751 20, Uppsala, Sweden

Email: karin.stensjo@kemi.uu.se

Supplementary Figure 1

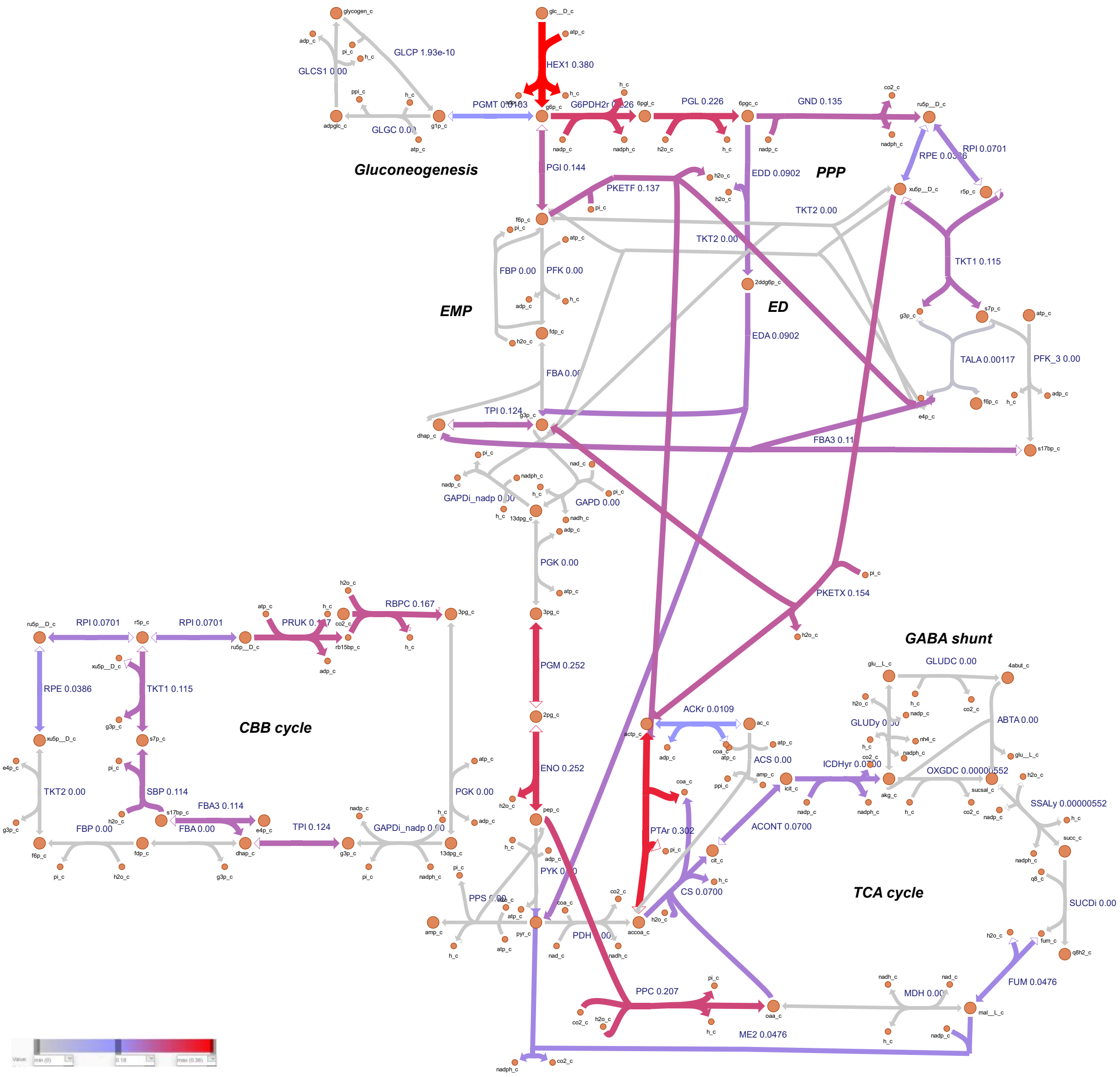

Supplementary Figure 1 Metabolic flux map of central carbon metabolism for *Synechocystis* sp. PCC 6803 with biomass set as objective, simulated to grow under mixotrophic conditions. Reaction fluxes (mmol/gDW/h) were predicted using pFBA[1]. Note that, the colors associated with the fluxes are relative to the other reactions rates presented in the map. Irreversible reactions are indicated by one-headed arrows; reversible reactions are indicated by two-headed arrows. For reaction directionality, refer to the data availability section. The map was generated with Escher web-tool[2]. Metabolic reactions and metabolites are indicated by their BiGG identifier[3].

Supplementary Figure 2

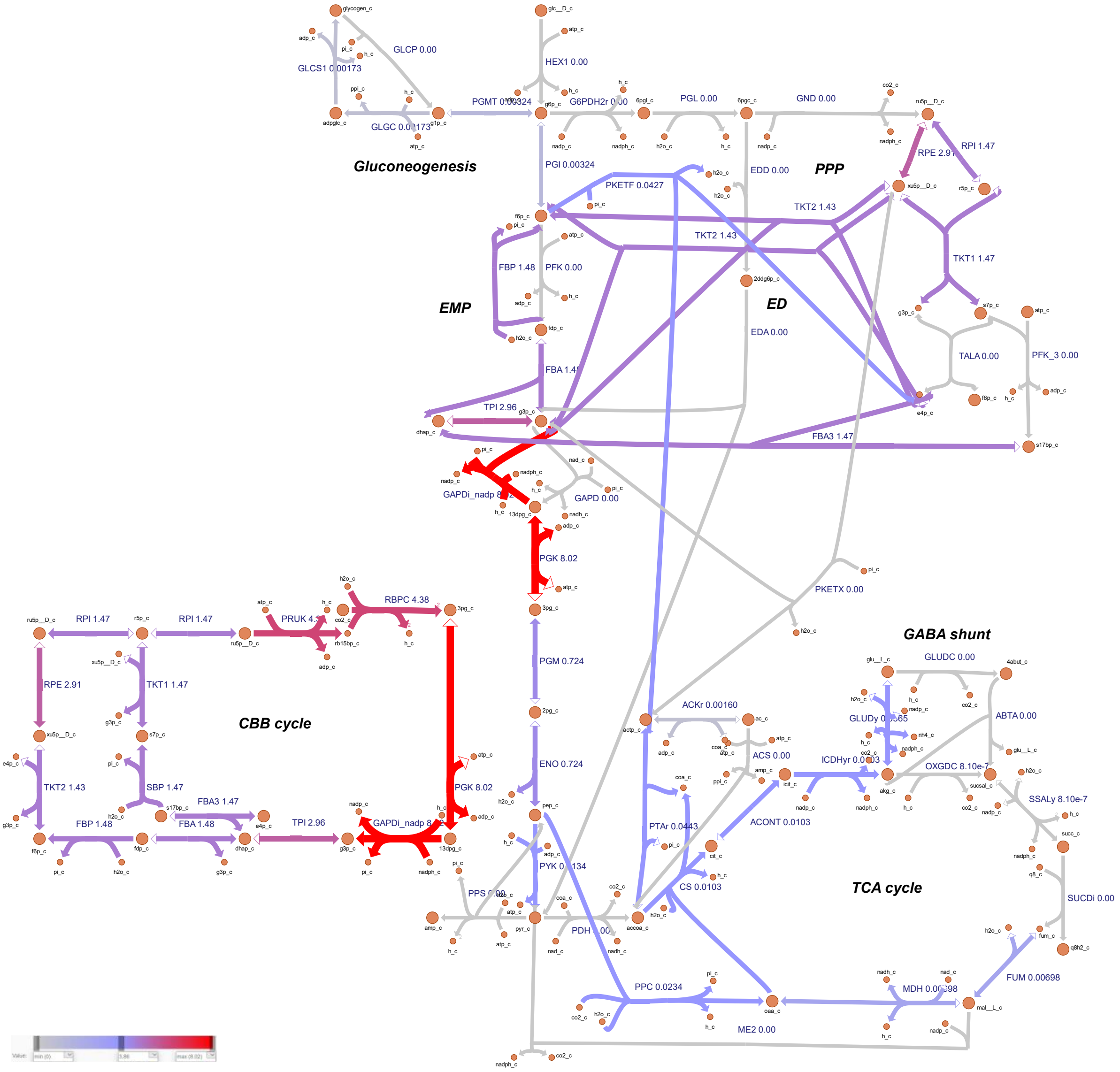

Supplementary Figure 2 Metabolic flux map of central carbon metabolism for *Synechocystis* sp. PCC 6803 with isoprene set as objective, simulated to grow under autotrophic conditions. Reaction fluxes (mmol/gDW/h) were predicted using pFBA[1]. Note that, the colors associated with the fluxes are relative to the other reactions rates presented in the map. Irreversible reactions are indicated by one-headed arrows; reversible reactions are indicated by two-headed arrows. For reaction directionality, refer to the data availability section. The map was generated with Escher web-tool[2]. Metabolic reactions and metabolites are indicated by their BiGG identifier[3].

Supplementary Figure 3

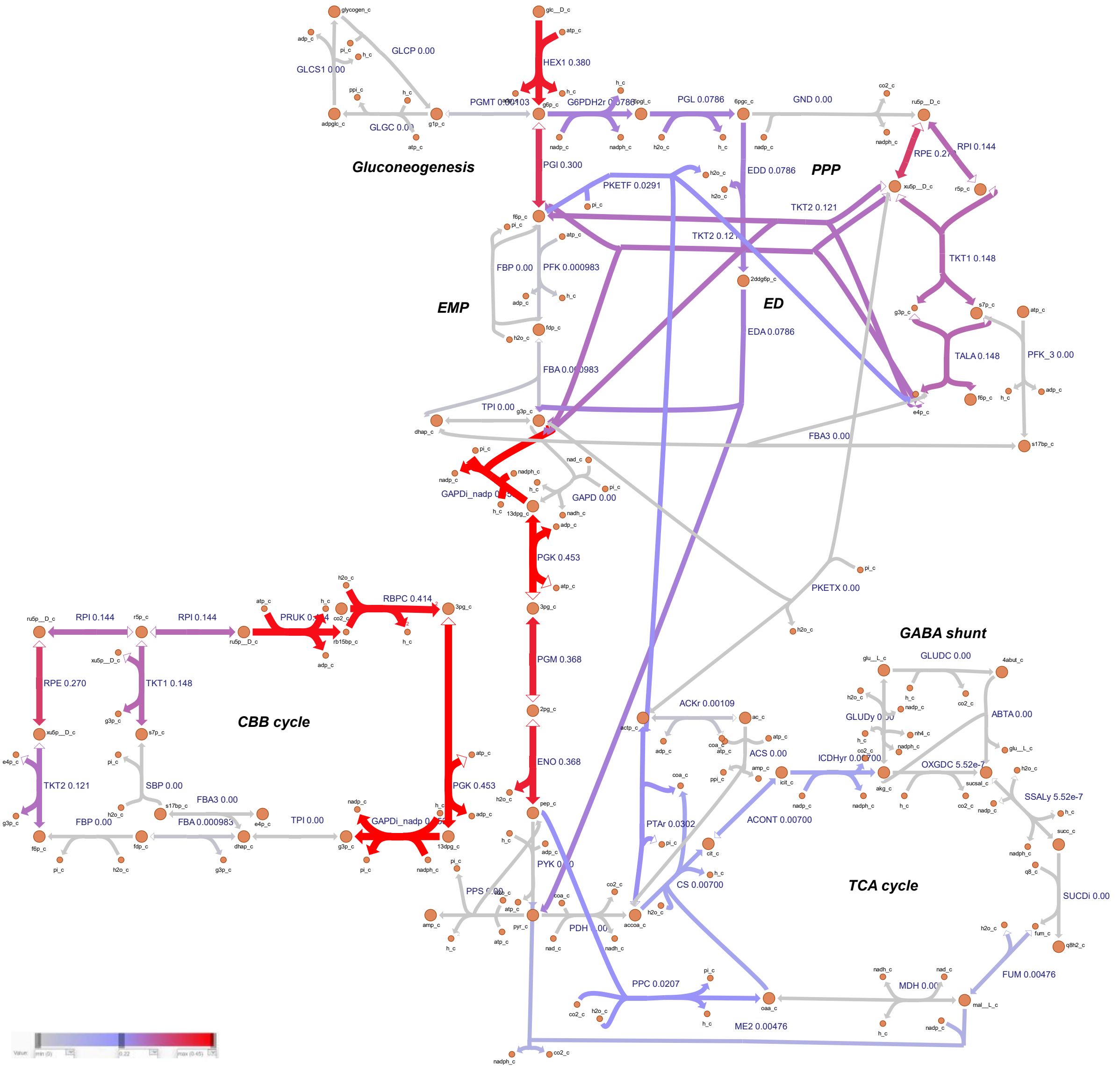

Supplementary Figure 3 Metabolic flux map of central carbon metabolism for *Synechocystis* sp. PCC 6803 with isoprene set as objective, simulated to grow under mixotrophic conditions. Reaction fluxes (mmol/gDW/h) were predicted using pFBA[1]. Note that, the colors associated with the fluxes are relative to the other reactions rates presented in the map. Irreversible reactions are indicated by one-headed arrows; reversible reactions are indicated by two-headed arrows. For reaction directionality, refer to the data availability section. The map was generated with Escher web-tool[2]. Metabolic reactions and metabolites are indicated by their BiGG identifier[3].

Supplementary Figure 4

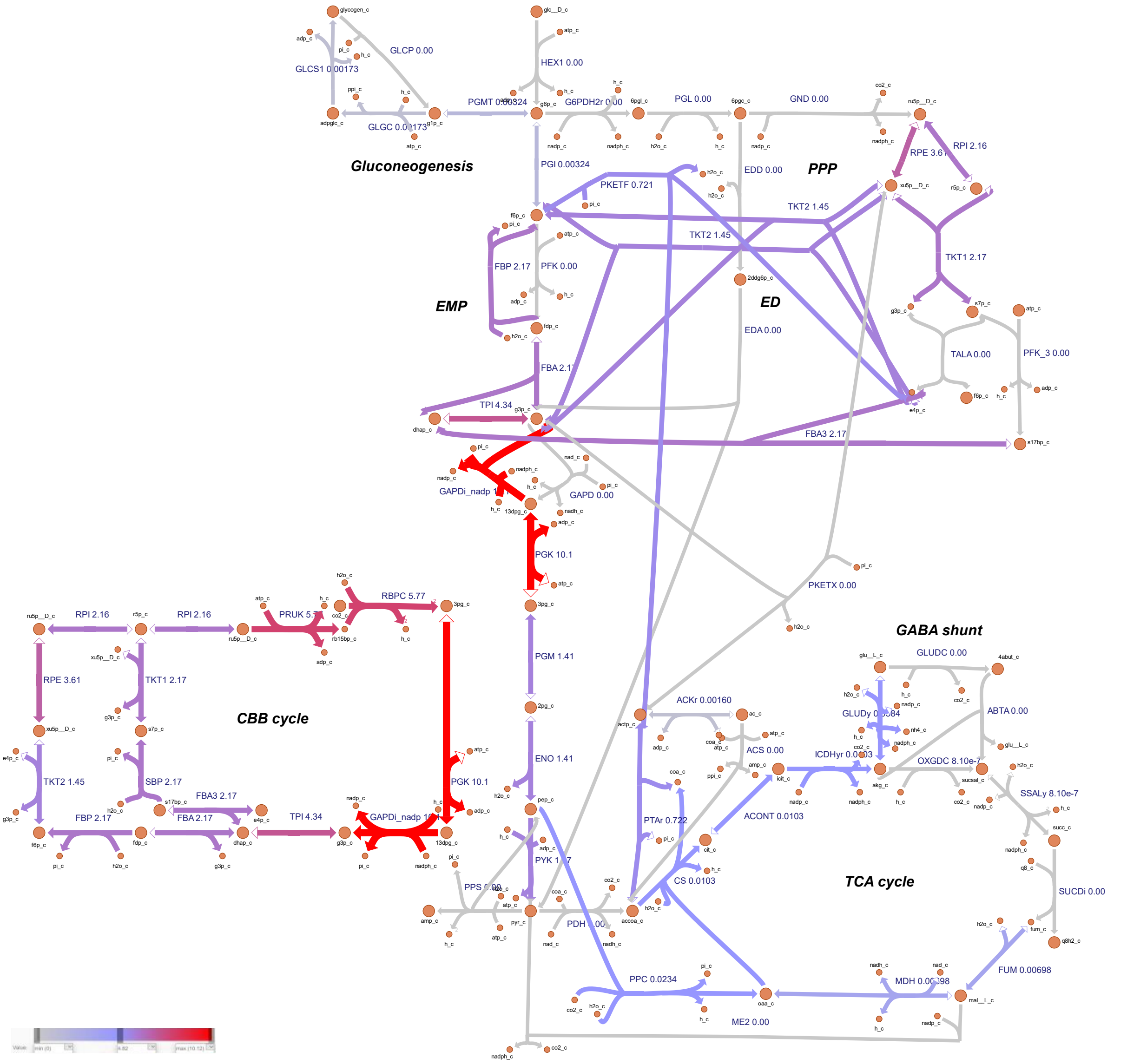

Supplementary Figure 4 Metabolic flux map of central carbon metabolism for *Synechocystis* sp. PCC 6803 with isobutene set as objective, simulated to grow under autotrophic conditions. Reaction fluxes (mmol/gDW/h) were predicted using pFBA[1]. Note that, the colors associated with the fluxes are relative to the other reactions rates presented in the map. Irreversible reactions are indicated by one-headed arrows; reversible reactions are indicated by two-headed arrows. For reaction directionality, refer to the data availability section. The map was generated with Escher web-tool[2]. Metabolic reactions and metabolites are indicated by their BiGG identifier[3].

Supplementary Figure 5

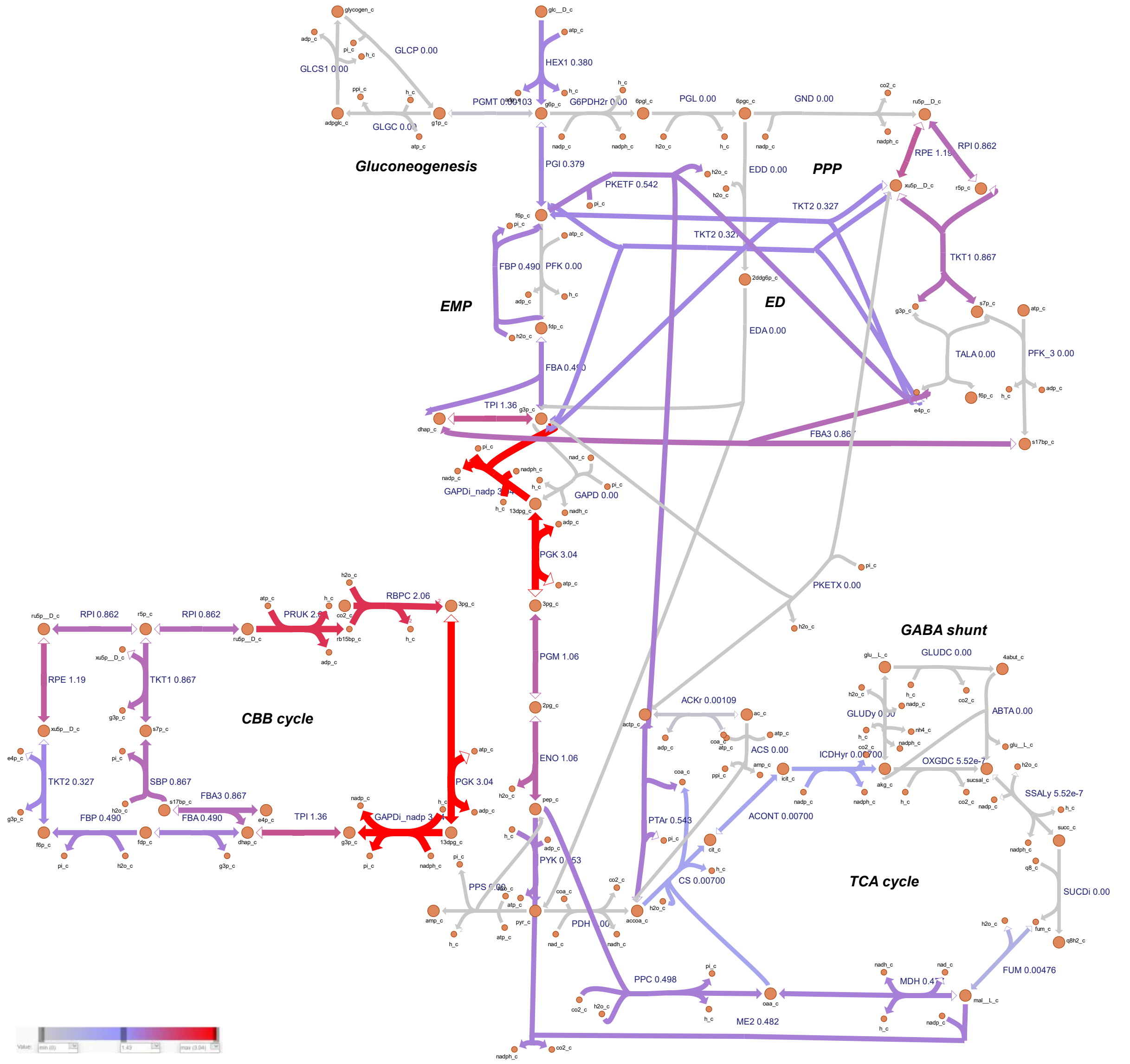

Supplementary Figure 5 Metabolic flux map of central carbon metabolism for *Synechocystis* sp. PCC 6803 with isobutene set as objective, simulated to grow under mixotrophic conditions. Reaction fluxes (mmol/gDW/h) were predicted using pFBA[1]. Note that, the colors associated with the fluxes are relative to the other reactions rates presented in the map. Irreversible reactions are indicated by one-headed arrows; reversible reactions are indicated by two-headed arrows. For reaction directionality, refer to the data availability section. The map was generated with Escher web-tool[2]. Metabolic reactions and metabolites are indicated by their BiGG identifier[3].

Supplementary Figure 6

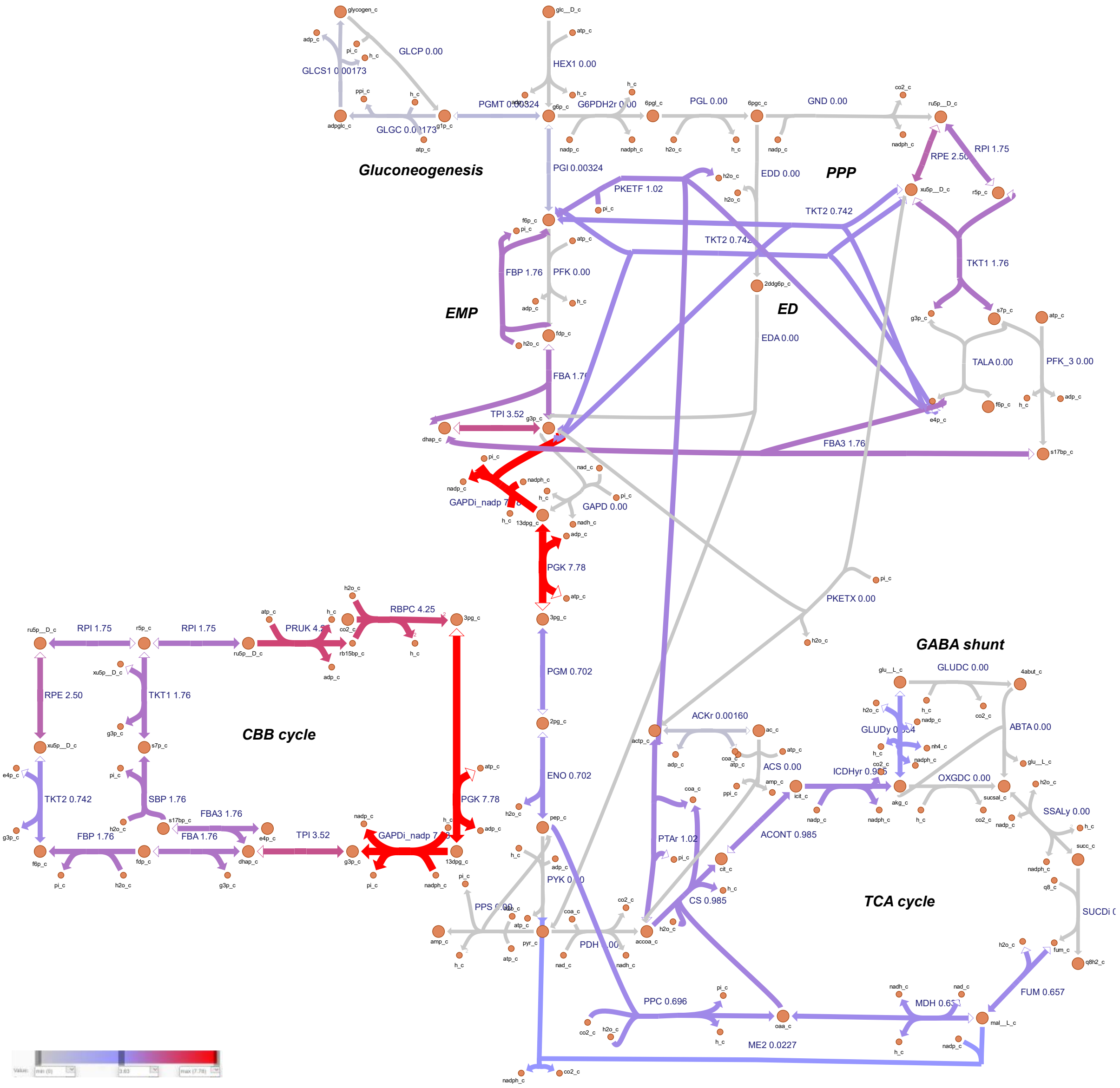

Supplementary Figure 6 Metabolic flux map of central carbon metabolism for *Synechocystis* sp. PCC 6803 with ethylene set as objective, simulated to grow under autotrophic conditions. Reaction fluxes (mmol/gDW/h) were predicted using pFBA[1]. Note that, the colors associated with the fluxes are relative to the other reactions rates presented in the map. Irreversible reactions are indicated by one-headed arrows; reversible reactions are indicated by two-headed arrows. For reaction directionality, refer to the data availability section. The map was generated with Escher web-tool[2]. Metabolic reactions and metabolites are indicated by their BiGG identifier[3].

Supplementary Figure 7

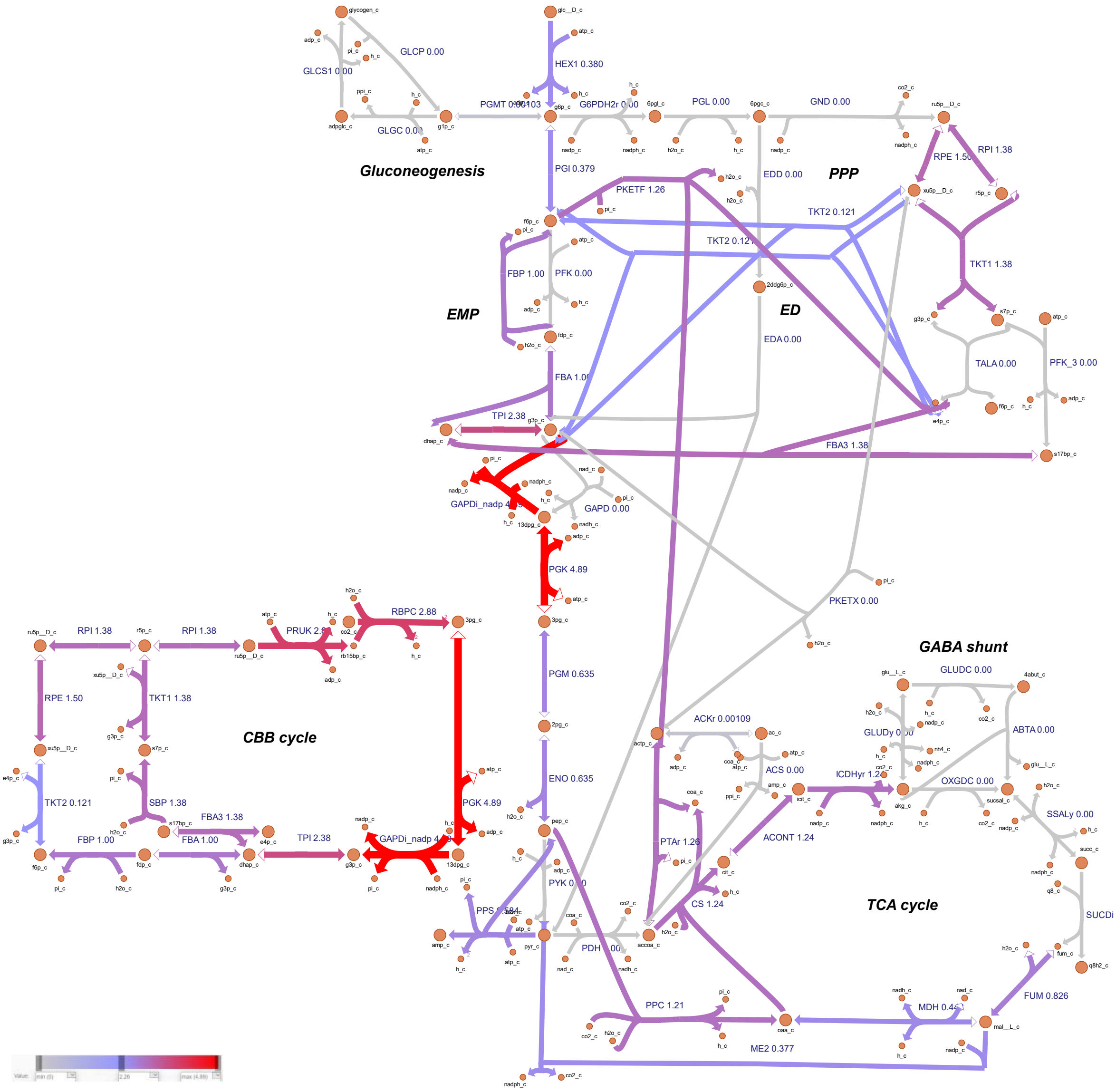

Supplementary Figure 7 Metabolic flux map of central carbon metabolism for *Synechocystis* sp. PCC 6803 with ethylene set as objective, simulated to grow under mixotrophic conditions. Reaction fluxes (mmol/gDW/h) were predicted using pFBA[1]. Note that, the colors associated with the fluxes are relative to the other reactions rates presented in the map. Irreversible reactions are indicated by one-headed arrows; reversible reactions are indicated by two-headed arrows. For reaction directionality, refer to the data availability section. The map was generated with Escher web-tool[2]. Metabolic reactions and metabolites are indicated by their BiGG identifier[3].

Supplementary Figure 8

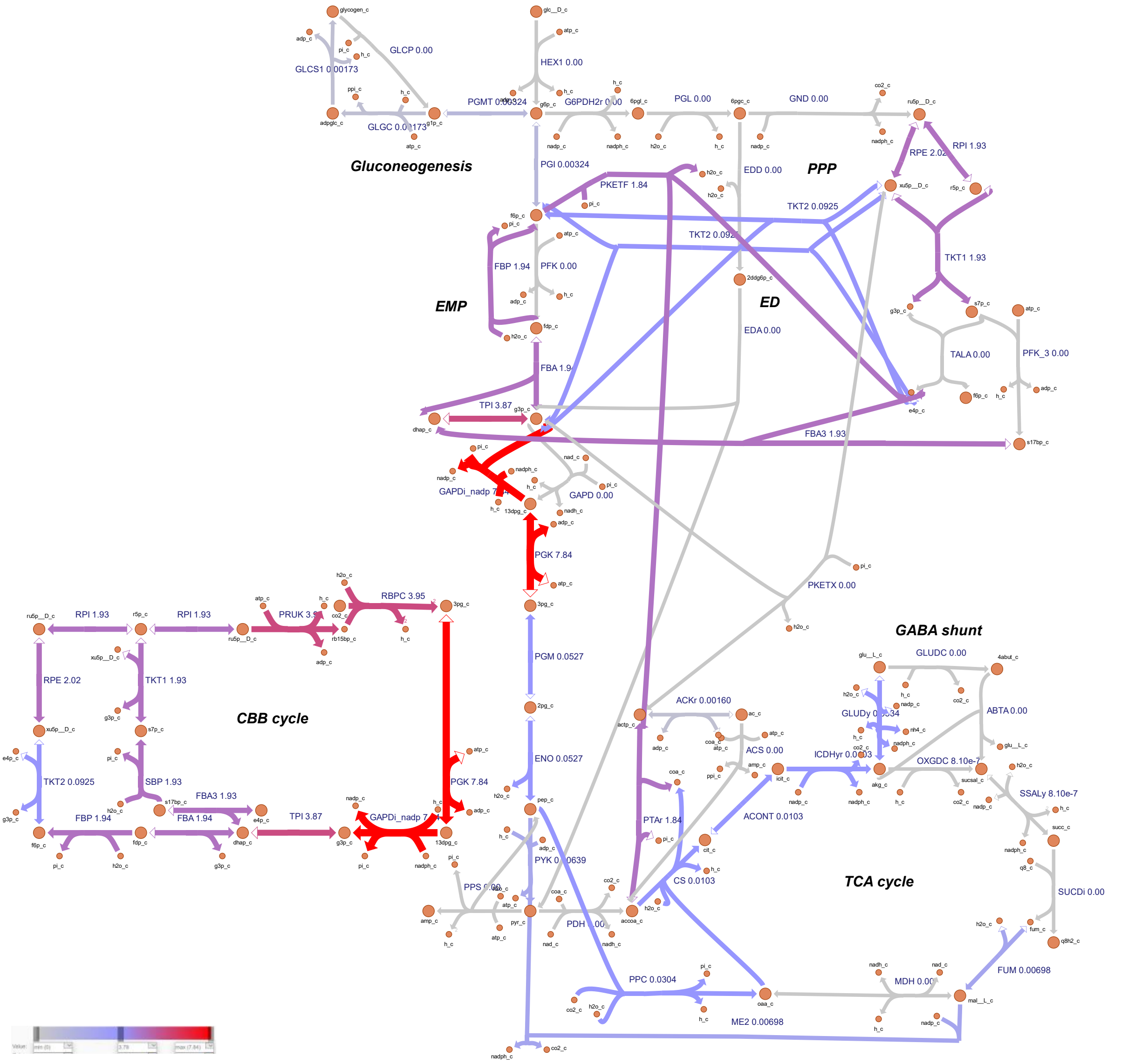

Supplementary Figure 8 Metabolic flux map of central carbon metabolism for *Synechocystis* sp. PCC 6803 with 1-undecene set as objective, simulated to grow under autotrophic conditions. Reaction fluxes (mmol/gDW/h) were predicted using pFBA[1]. Note that, the colors associated with the fluxes are relative to the other reactions rates presented in the map. Irreversible reactions are indicated by one-headed arrows; reversible reactions are indicated by two-headed arrows. For reaction directionality, refer to the data availability section. The map was generated with Escher web-tool[2]. Metabolic reactions and metabolites are indicated by their BiGG identifier[3].

Supplementary Figure 9

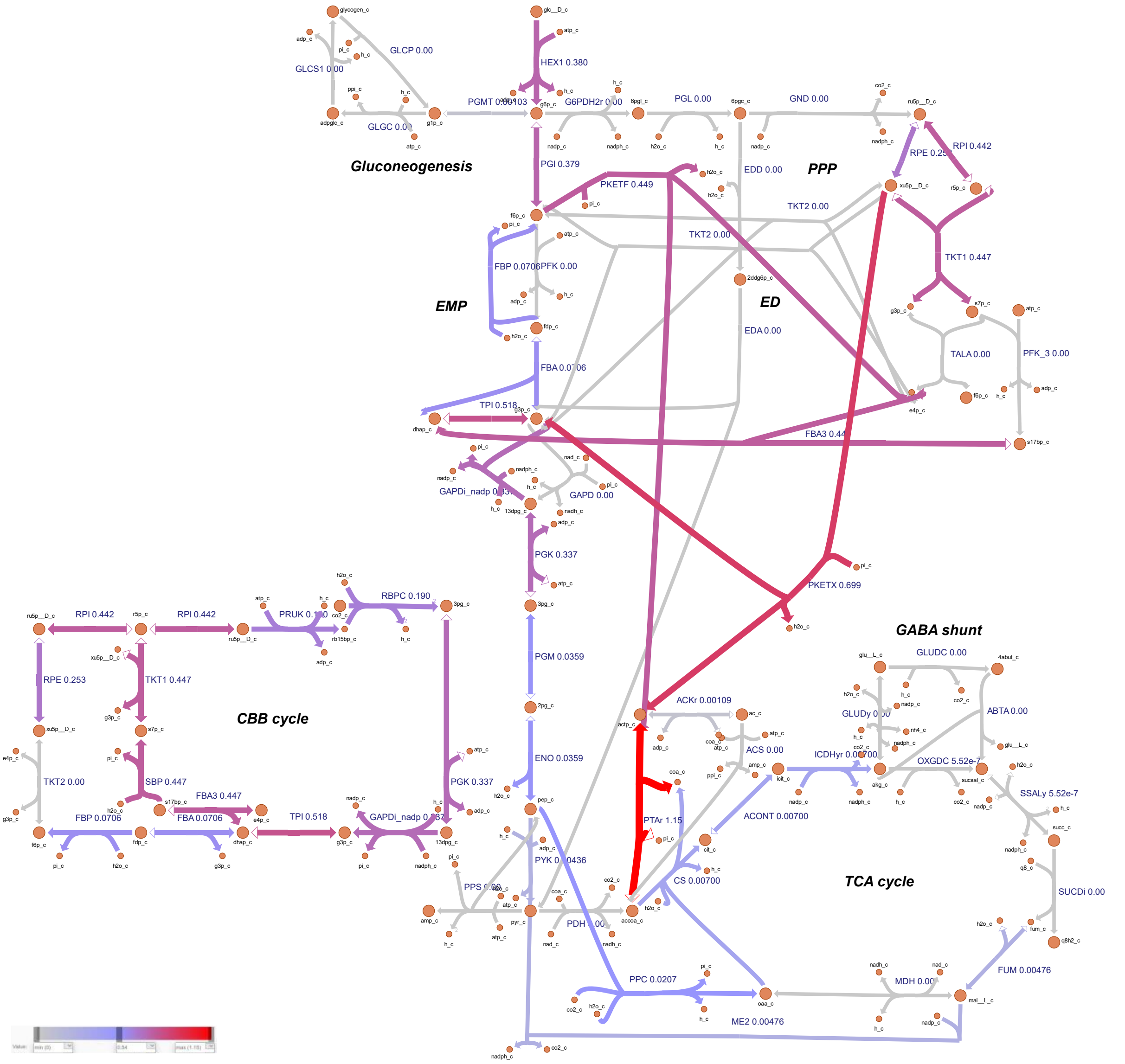

Supplementary Figure 9 Metabolic flux map of central carbon metabolism for *Synechocystis* sp. PCC 6803 with 1-undecene set as objective, simulated to grow under mixotrophic conditions. Reaction fluxes (mmol/gDW/h) were predicted using pFBA[1]. Note that, the colors associated with the fluxes are relative to the other reactions rates presented in the map. Irreversible reactions are indicated by one-headed arrows; reversible reactions are indicated by two-headed arrows. For reaction directionality, refer to the data availability section. The map was generated with Escher web-tool[2]. Metabolic reactions and metabolites are indicated by their BiGG identifier[3].

Supplementary Figure 10

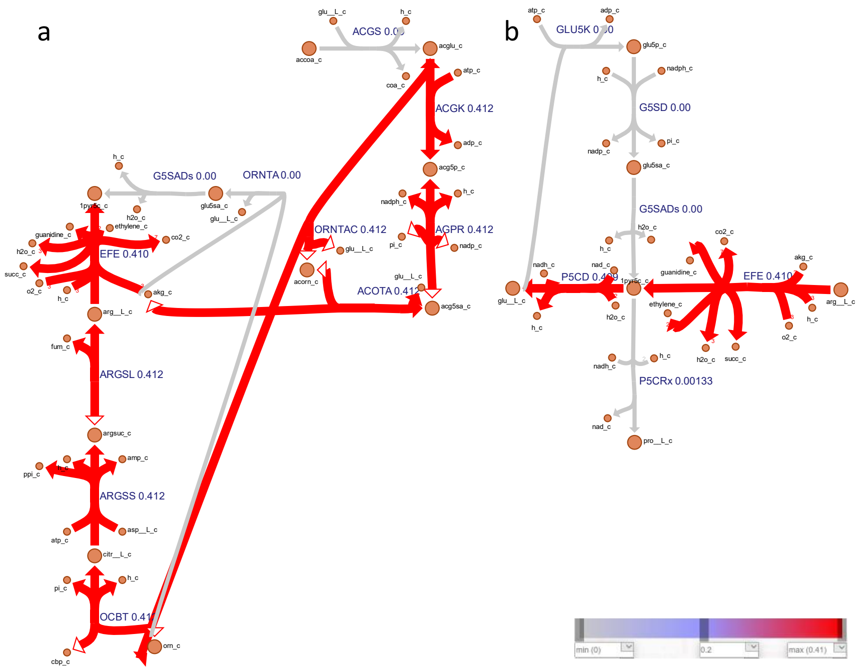

Supplementary Figure 10 Metabolic flux map of nitrogen metabolism for *Synechocystis* sp. PCC 6803 with ethylene set as objective, simulated to grow under mixotrophic conditions. (A) L-glutamate and L-arginine regeneration through the urea cycle. (B) L-glutamate and L-proline regeneration. Reaction fluxes (mmol/gDW/h) were predicted using pFBA[1]. Note that, the colors associated with the fluxes are relative to the other reactions rates presented in the map. Irreversible reactions are indicated by one-headed arrows; reversible reactions are indicated by two-headed arrows. For reaction directionality, refer to the data availability section. The map was generated with Escher web-tool[2]. Metabolic reactions and metabolites are indicated by their BiGG identifier[3].

Supplementary Figure 11

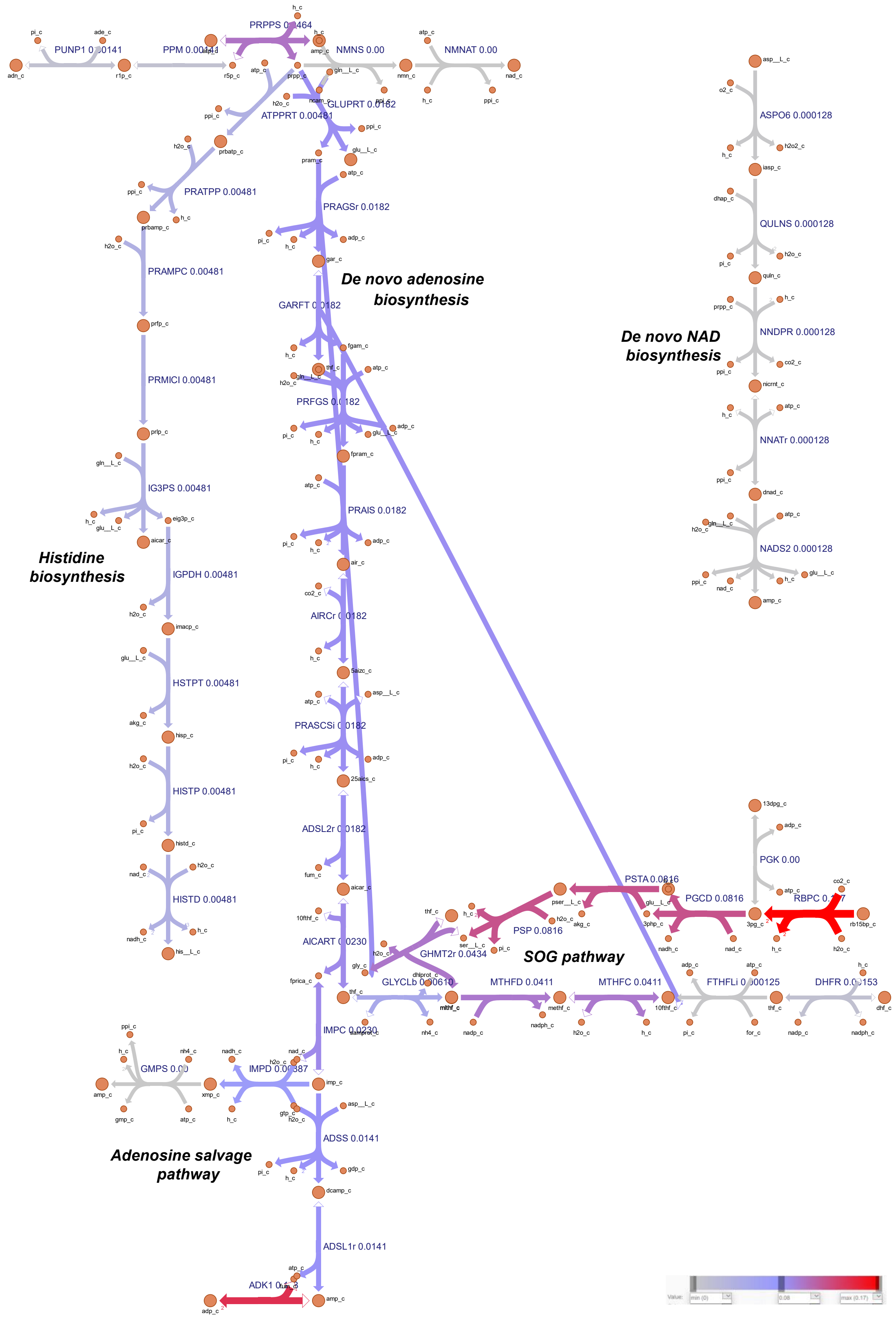

Supplementary Figure 11 Metabolic flux map of energy and redox metabolism for *Synechocystis* sp. PCC 6803 with biomass set as objective, simulated to grow under mixotrophic conditions. Reaction fluxes (mmol/gDW/h) were predicted using pFBA[1]. Note that, the colors associated with the fluxes are relative to the other reactions rates presented in the map. Irreversible reactions are indicated by one-headed arrows; reversible reactions are indicated by two-headed arrows. For reaction directionality, refer to the data availability section. The map was generated with Escher web-tool[2]. Metabolic reactions and metabolites are indicated by their BiGG identifier[3].

Supplementary Figure 12

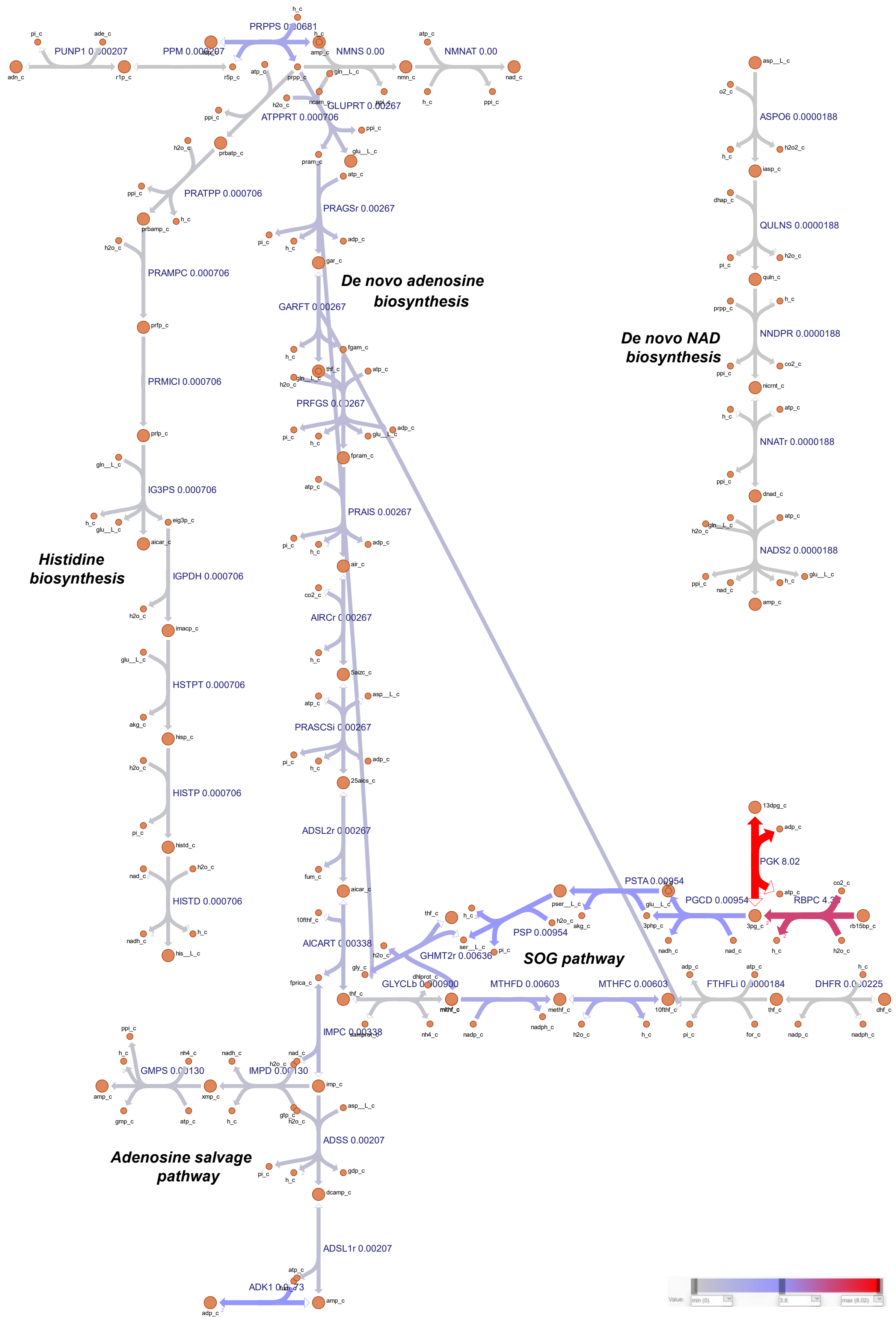

Supplementary Figure 12 Metabolic flux map of energy and redox metabolism for *Synechocystis* sp. PCC 6803 with isoprene set as objective, simulated to grow under autotrophic conditions. Reaction fluxes (mmol/gDW/h) were predicted using pFBA[1]. Note that, the colors associated with the fluxes are relative to the other reactions rates presented in the map. Irreversible reactions are indicated by one-headed arrows; reversible reactions are indicated by two-headed arrows. For reaction directionality, refer to the data availability section. The map was generated with Escher web-tool[2]. Metabolic reactions and metabolites are indicated by their BiGG identifier[3].

Supplementary Figure 13

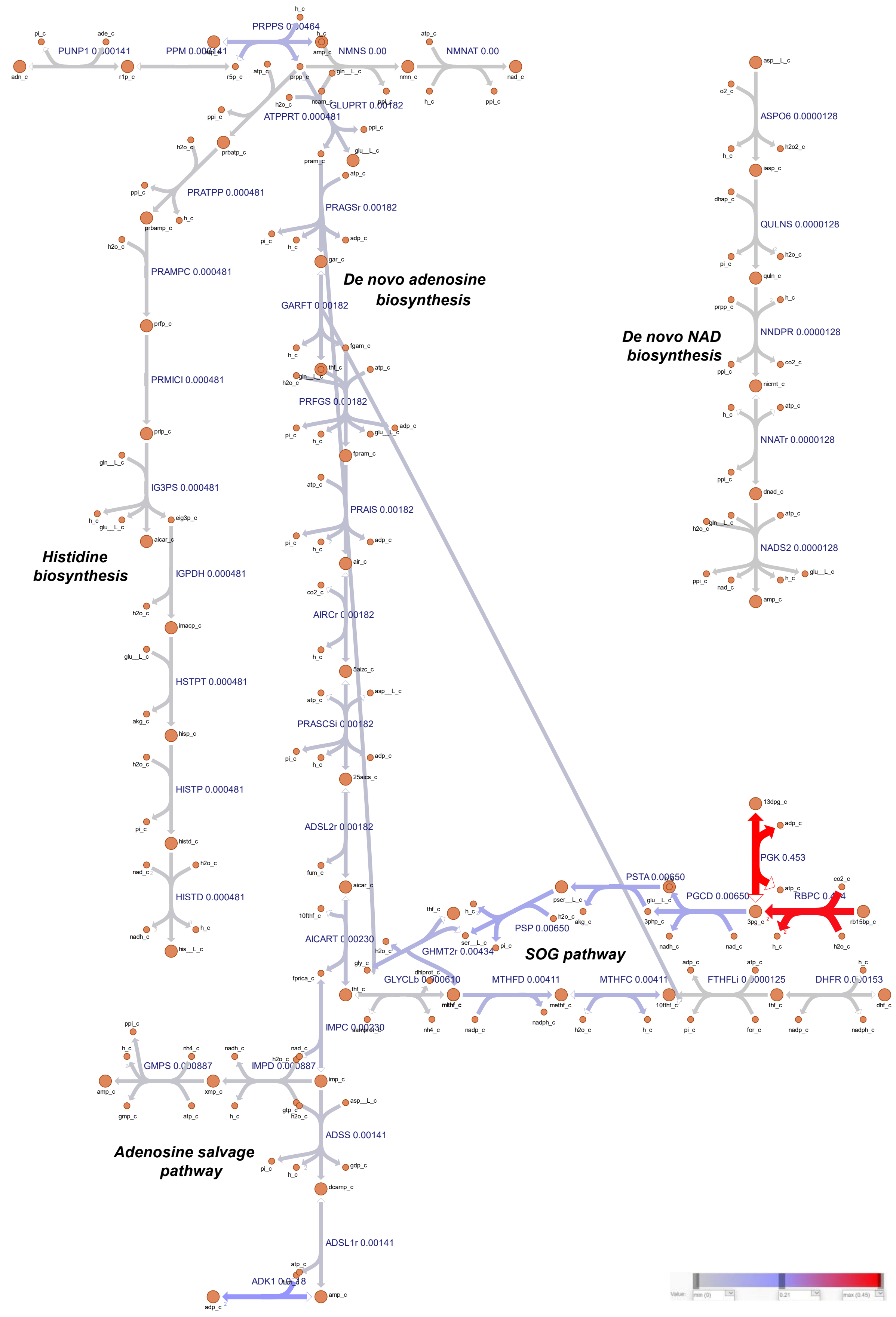

Supplementary Figure 13 Metabolic flux map of energy and redox metabolism for *Synechocystis* sp. PCC 6803 with isoprene set as objective, simulated to grow under mixotrophic conditions. Reaction fluxes (mmol/gDW/h) were predicted using pFBA[1]. Note that, the colors associated with the fluxes are relative to the other reactions rates presented in the map. Irreversible reactions are indicated by one-headed arrows; reversible reactions are indicated by two-headed arrows. For reaction directionality, refer to the data availability section. The map was generated with Escher web-tool[2]. Metabolic reactions and metabolites are indicated by their BiGG identifier[3].

Supplementary Figure 14

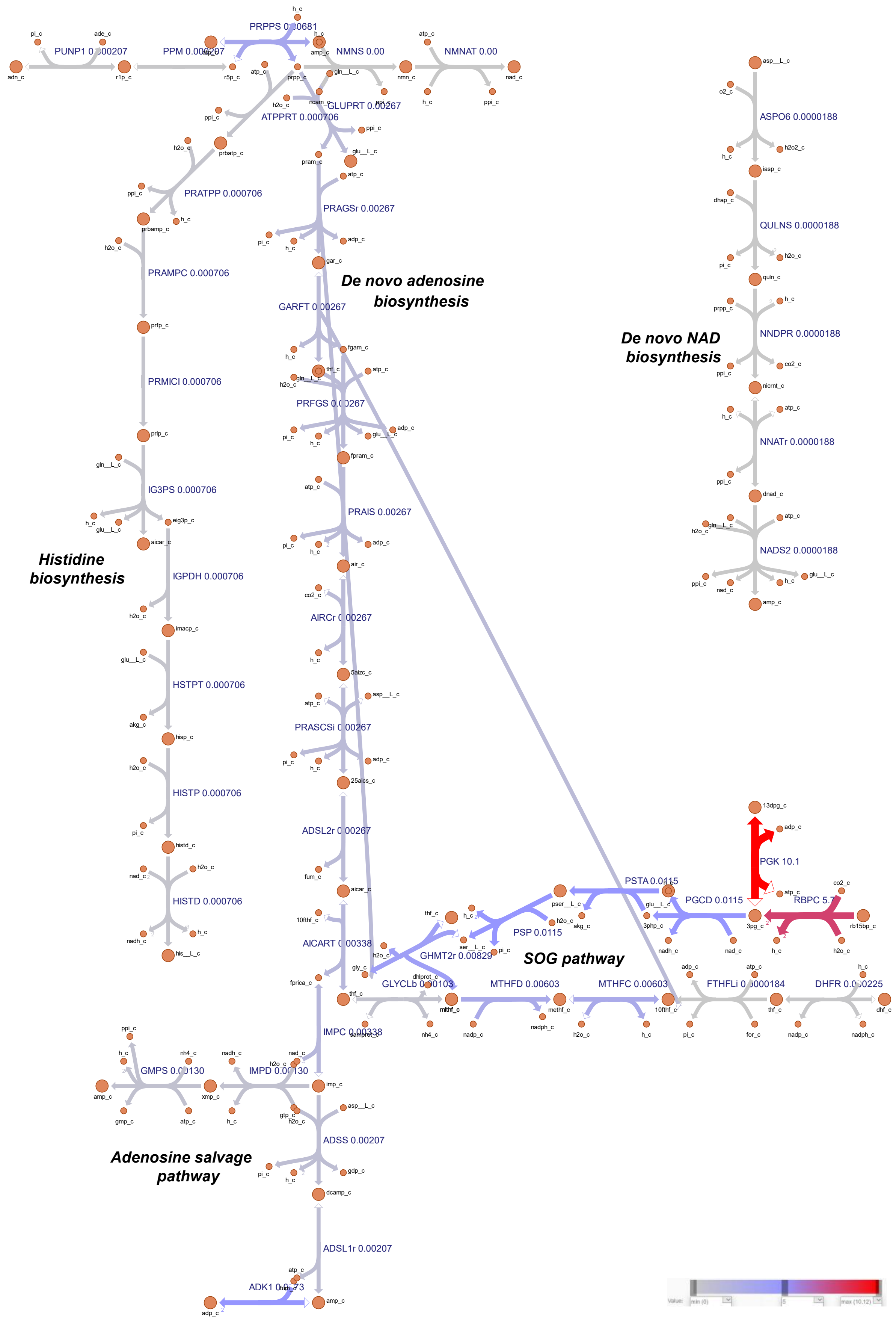

Supplementary Figure 14 Metabolic flux map of energy and redox metabolism for *Synechocystis* sp. PCC 6803 with isobutene set as objective, simulated to grow under autotrophic conditions. Reaction fluxes (mmol/gDW/h) were predicted using pFBA[1]. Note that, the colors associated with the fluxes are relative to the other reactions rates presented in the map. Irreversible reactions are indicated by one-headed arrows; reversible reactions are indicated by two-headed arrows. For reaction directionality, refer to the data availability section. The map was generated with Escher web-tool[2]. Metabolic reactions and metabolites are indicated by their BiGG identifier[3].

Supplementary Figure 15

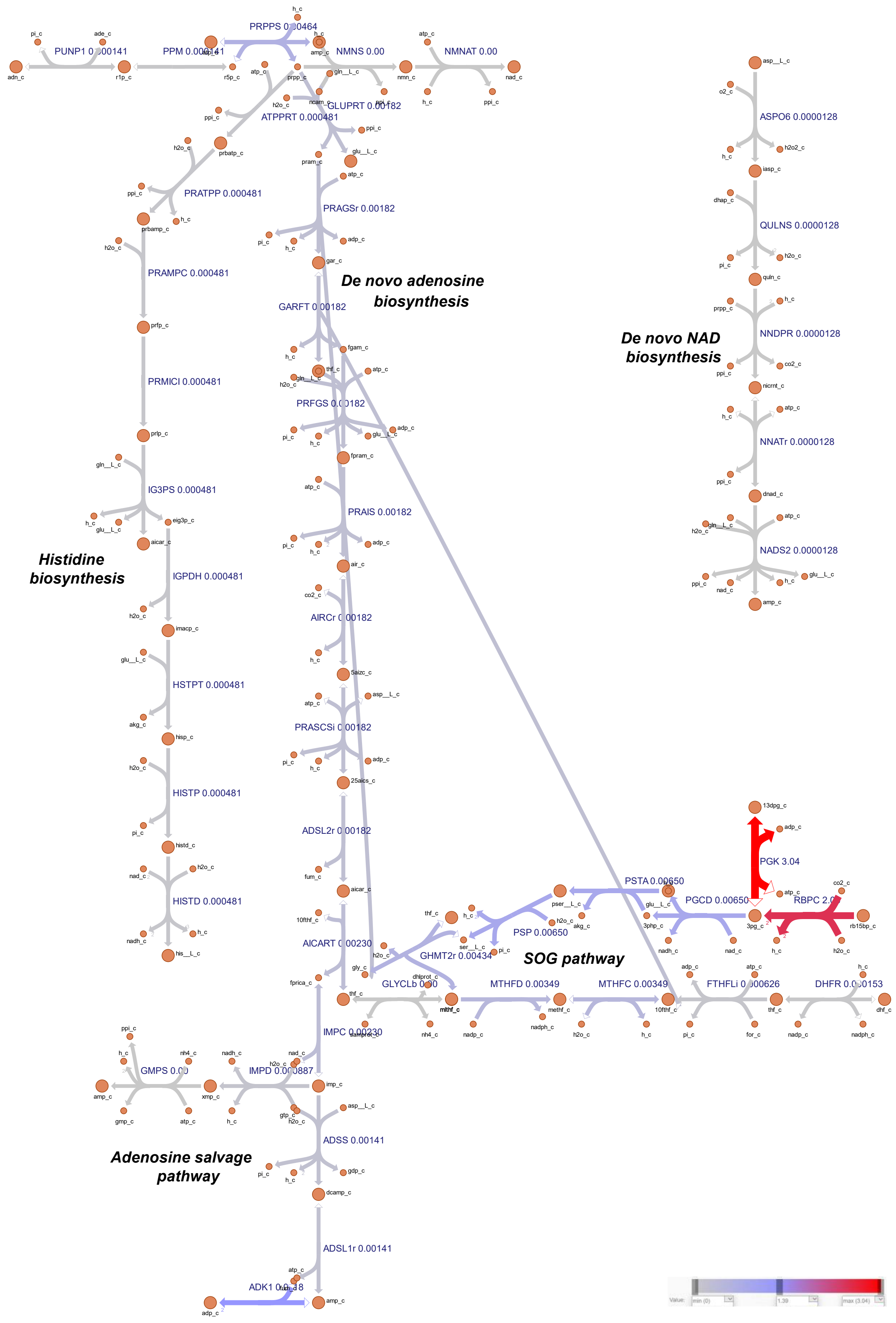

Supplementary Figure 15 Metabolic flux map of energy and redox metabolism for *Synechocystis* sp. PCC 6803 with isobutene set as objective, simulated to grow under mixotrophic conditions. Reaction fluxes (mmol/gDW/h) were predicted using pFBA[1]. Note that, the colors associated with the fluxes are relative to the other reactions rates presented in the map. Irreversible reactions are indicated by one-headed arrows; reversible reactions are indicated by two-headed arrows. For reaction directionality, refer to the data availability section. The map was generated with Escher web-tool[2]. Metabolic reactions and metabolites are indicated by their BiGG identifier[3].

Supplementary Figure 16

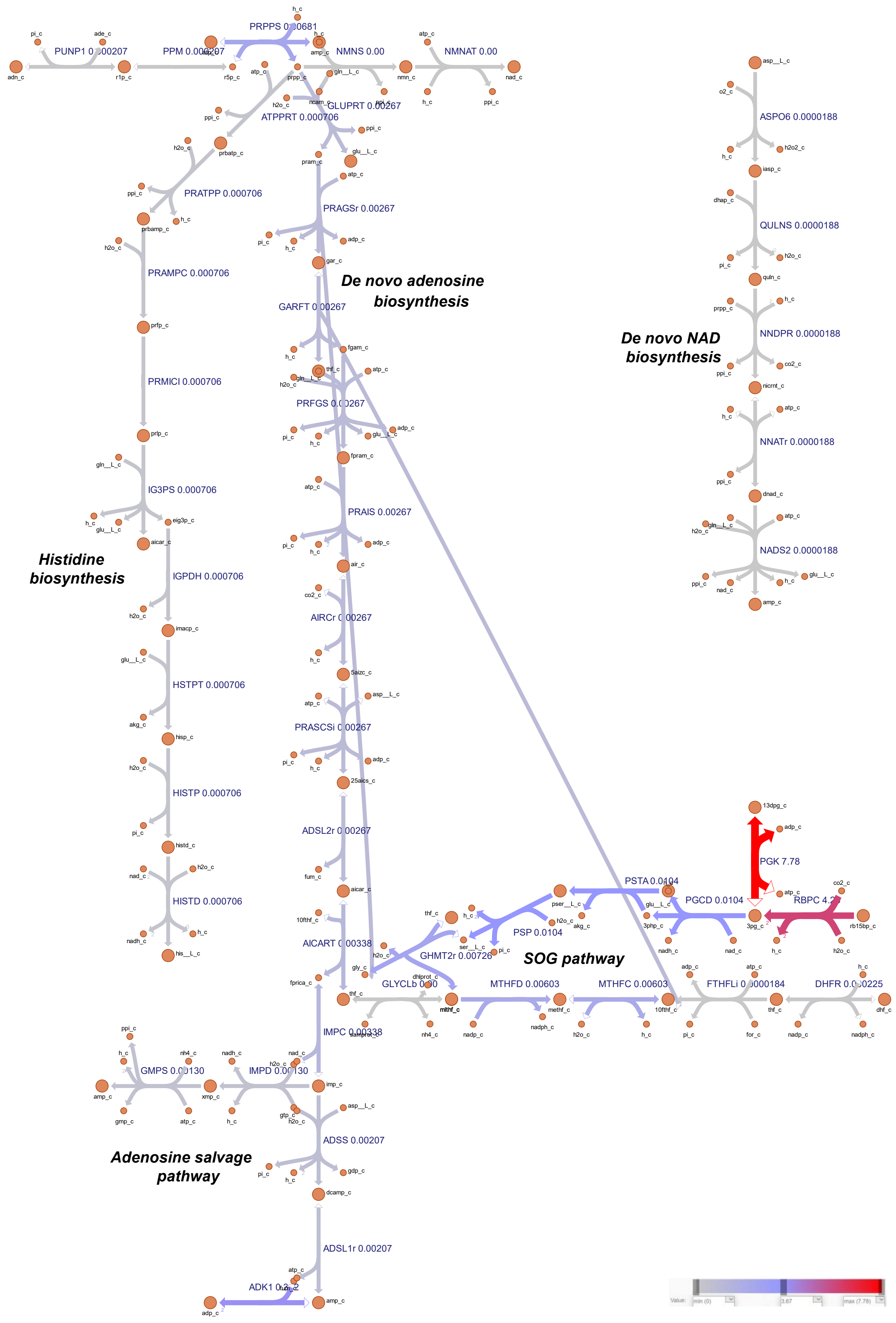

Supplementary Figure 16 Metabolic flux map of energy and redox metabolism for *Synechocystis* sp. PCC 6803 with ethylene set as objective, simulated to grow under autotrophic conditions. Reaction fluxes (mmol/gDW/h) were predicted using pFBA[1]. Note that, the colors associated with the fluxes are relative to the other reactions rates presented in the map. Irreversible reactions are indicated by one-headed arrows; reversible reactions are indicated by two-headed arrows. For reaction directionality, refer to the data availability section. The map was generated with Escher web-tool[2]. Metabolic reactions and metabolites are indicated by their BiGG identifier[3].

Supplementary Figure 17

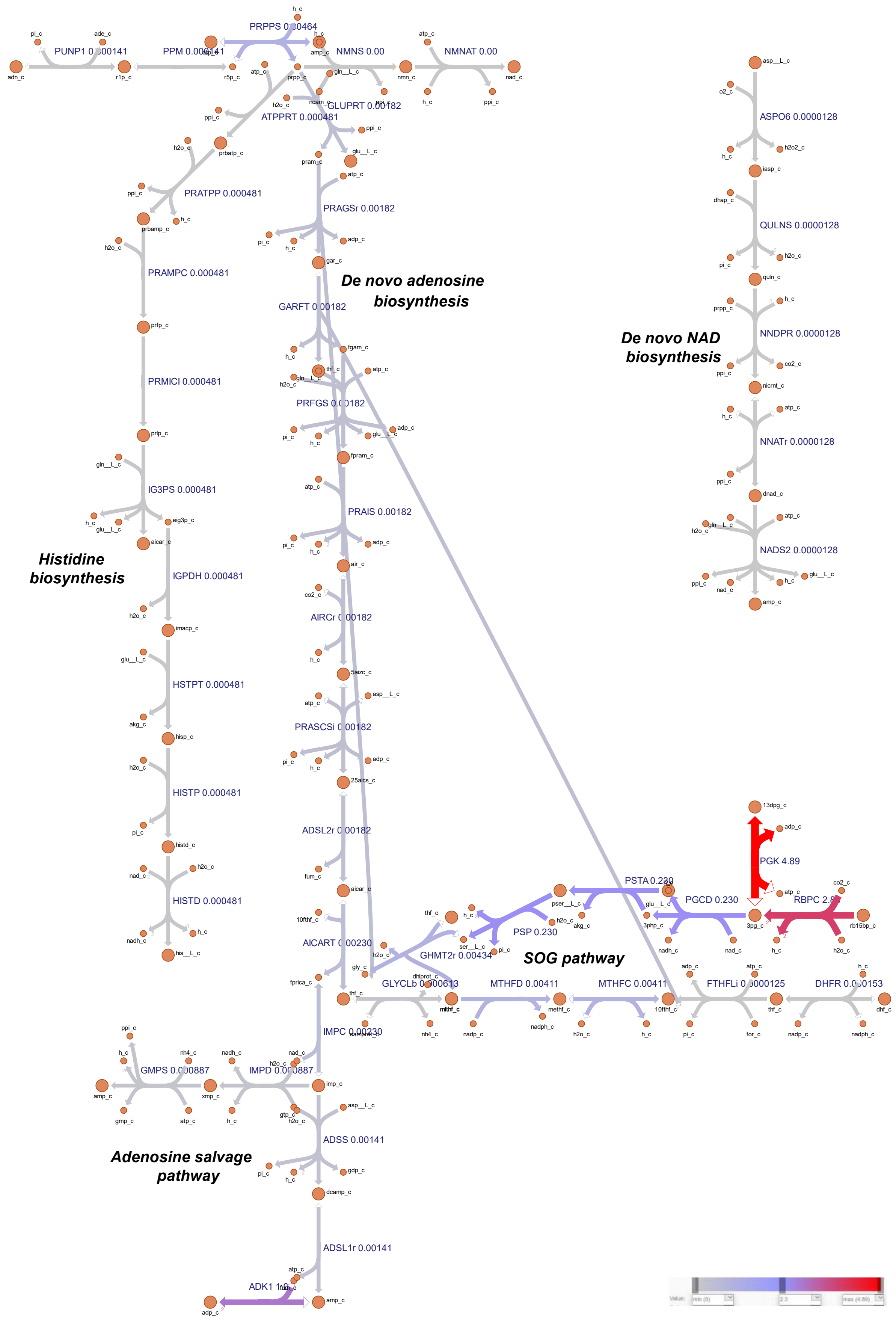

Supplementary Figure 17 Metabolic flux map of energy and redox metabolism for *Synechocystis* sp. PCC 6803 with ethylene set as objective, simulated to grow under mixotrophic conditions. Reaction fluxes (mmol/gDW/h) were predicted using pFBA[1]. Note that, the colors associated with the fluxes are relative to the other reactions rates presented in the map. Irreversible reactions are indicated by one-headed arrows; reversible reactions are indicated by two-headed arrows. For reaction directionality, refer to the data availability section. The map was generated with Escher web-tool[2]. Metabolic reactions and metabolites are indicated by their BiGG identifier[3].

Supplementary Figure 18

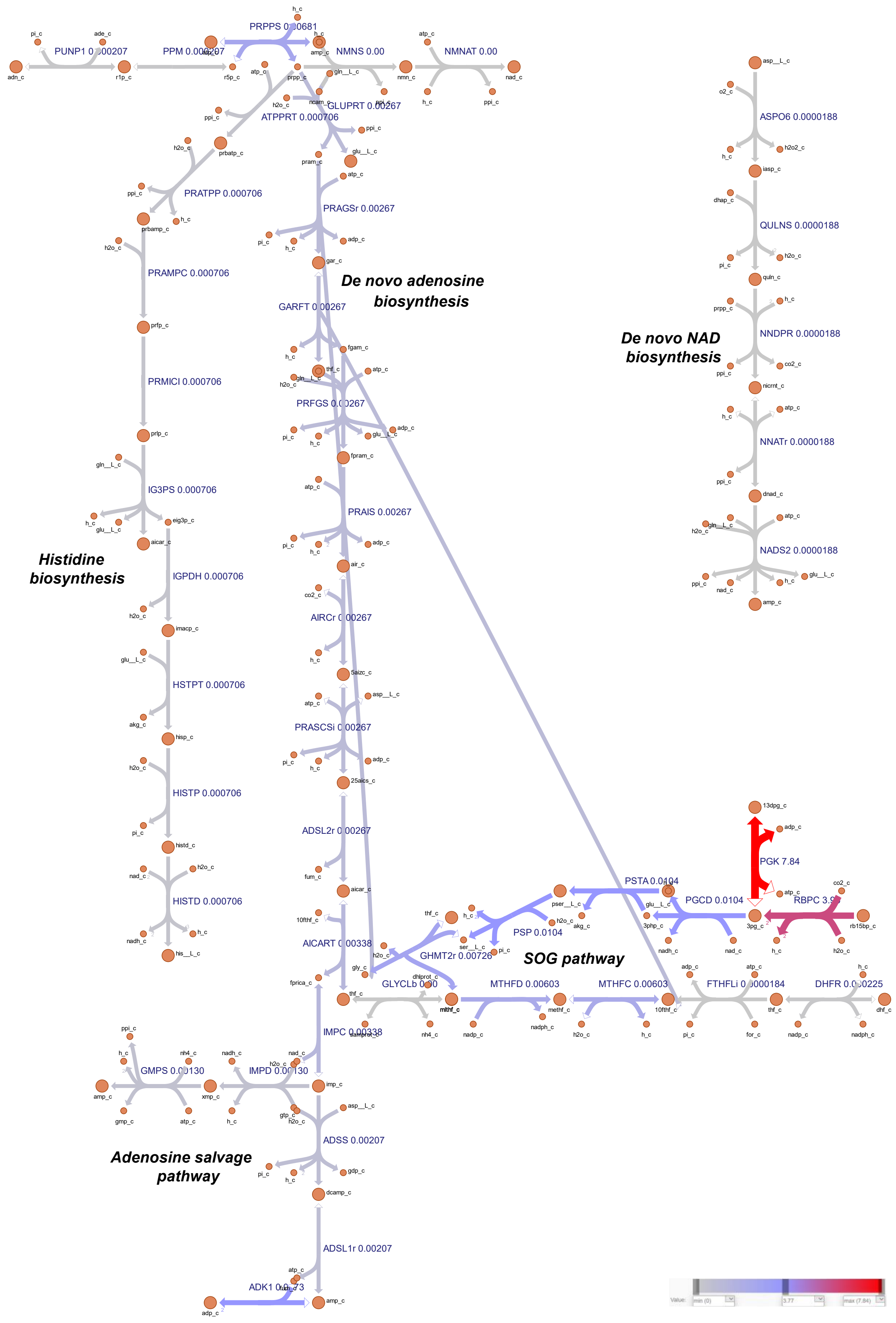

Supplementary Figure 18 Metabolic flux map of energy and redox metabolism for *Synechocystis* sp. PCC 6803 with 1-undecene set as objective, simulated to grow under autotrophic conditions. Reaction fluxes (mmol/gDW/h) were predicted using pFBA[1]. Note that, the colors associated with the fluxes are relative to the other reactions rates presented in the map. Irreversible reactions are indicated by one-headed arrows; reversible reactions are indicated by two-headed arrows. For reaction directionality, refer to the data availability section. The map was generated with Escher web-tool[2]. Metabolic reactions and metabolites are indicated by their BiGG identifier[3].

Supplementary Figure 19

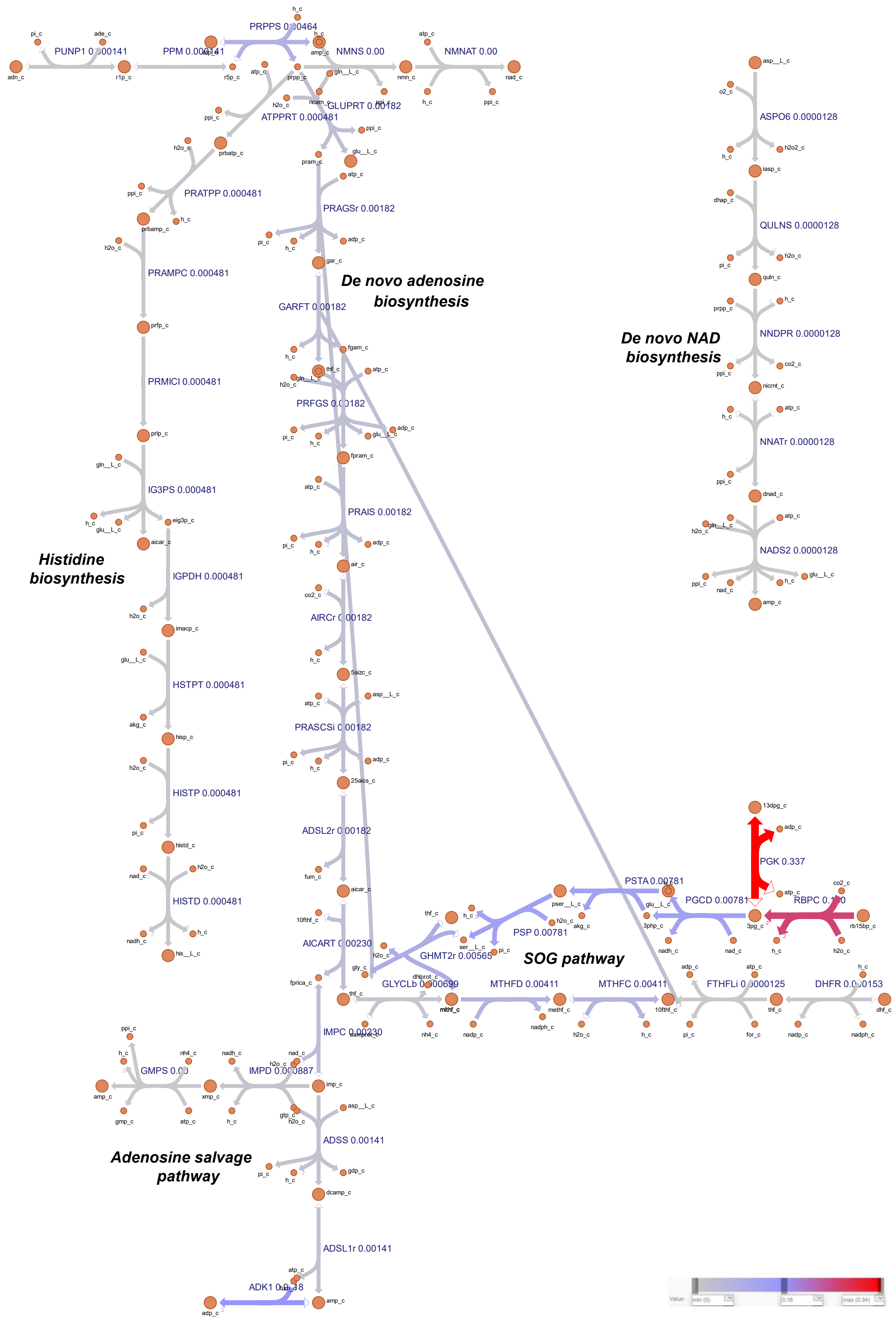

Supplementary Figure 19 Metabolic flux map of energy and redox metabolism for *Synechocystis* sp. PCC 6803 with 1-undecene set as objective, simulated to grow under mixotrophic conditions. Reaction fluxes (mmol/gDW/h) were predicted using pFBA[1]. Note that, the colors associated with the fluxes are relative to the other reactions rates presented in the map. Irreversible reactions are indicated by one-headed arrows; reversible reactions are indicated by two-headed arrows. For reaction directionality, refer to the data availability section. The map was generated with Escher web-tool[2]. Metabolic reactions and metabolites are indicated by their BiGG identifier[3].

References

1. Lewis, N. E. *et al.* Omic data from evolved *E. coli* are consistent with computed optimal growth from genome-scale models. *Mol. Syst. Biol.* **6**, 390 (2010).
2. King, Z. A. *et al.* Escher: A Web Application for Building, Sharing, and Embedding Data-Rich Visualizations of Biological Pathways. *PLOS Comput. Biol.* **11**, e1004321 (2015).
3. King, Z. A. *et al.* BiGG Models: A platform for integrating, standardizing and sharing genome-scale models. *Nucleic Acids Res.* **44**, D515–D522 (2016).

Supplementary material (tables) for Optimal energy and redox metabolism in the cyanobacterium *Synechocystis* sp. PCC 6803

Amit Kugler[1], Karin Stensjö[1]*

[1]Microbial Chemistry, Department of Chemistry-Ångström Laboratory, Uppsala University, Box 523, SE-751 20, Uppsala, Sweden

Email: karin.stensjo@kemi.uu.se

Supplementary Table 1 Reactions added to the iJN678 model to create iJN678_AK.

Name	Gene–Protein-Reaction Rule	Description	Stoichiometry	Reference
OXGDC	sll1981	2-oxoglutarate decarboxylase	akg_c + h_c --> co2_c + sucsal_c	[1–3]
PKETF	slr0453	Phosphoketolase (fructose-6-phosphate utilizing)	f6p_c + pi_c --> actp_c + e4p_c + h2o_c	[4,5]
PKETX	slr0453	Phosphoketolase (xylulose-5-phosphate utilizing)	pi_c + xu5p__D_c --> actp_c + g3p_c + h2o_c	[4,5]
GDH	sll1709	Glucose dehydrogenase	glc__bD_c + h2o_c + nadp_c --> glcn_c + 2 h_c + nadph_c	[6]
GNK	sll0593	Gluconokinase	atp_c + glcn_c --> 6pgc_c + adp_c + h_c	[6]
EDD	slr0452	6-phosphogluconate dehydratase	6pgc_c --> 2ddg6p_c + h2o_c	[6]
EDA	sll0107	2-dehydro-3-deoxy-phosphogluconate aldolase	2ddg6p_c --> g3p_c + pyr_c	[6]
PSTA	sll1559	Phosphoserine transaminase	3php_c + glu__L_c --> akg_c + pser__L_c	[7]
PSP	slr1124	Phosphoserine phosphatase	h2o_c + pser__L_c --> 2 h_c + pi_c + ser__L_c	[7]
PAT1	Not known	Prephenate aminotransferase (aspartate donor)	asp__L_c + h_c + pphn_c --> Largn_c + oaa_c	[8]
PAT2	Not known	Prephenate aminotransferase (glutamate donor)	glu__L_c + h_c + pphn_c --> Largn_c + akg_c	[8]
PHEA	sll1662	Prephenate dehydratase	Largn_c --> co2_c + h2o_c + phe__L_c	[8]
TYRA	slr2081	Arogenate dehydrogenase	Largn_c + nadp_c --> co2_c + h_c + nadph_c + tyr__L_c	[8]
GLYCL	sll0171, slr0879, slr0293	Glycine Cleavage System	Fixed to zero	[9]
GLYCL_2	sll0171, slr0879, slr0293	Glycine Cleavage System	Fixed to zero	[9]

GLYCLa	sll0171, slr0879, slr0293	Glycine Cleavage System	gly_c + lprot_c ⇌ co2_c + samprot_c	[9]
GLYCLb	sll0171, slr0879, slr0293	Glycine Cleavage System	thf_c + samprot_c ⇌ mlthf_c + nh4_c + dhlprot_c	[9]
GLYCLc	sll0171, slr0879, slr0293	Glycine Cleavage System	nad_c + dhlprot_c ⇌ h_c + nadh_c + lprot_c	[9]
NADTRHD	slr1239, slr1434	NAD transhydrogenase	nad_c + nadph_c ⇌ nadh_c + nadp_c	[10]
NDH2_1p	slr1743	NADH dehydrogenase 2	h_c + nadh_c + pq_p → nad_c + pqh2_p	[11,12]
ARTO	slr2082 slr2083 sll0813	Alternative respiratory terminal oxidase	2 h_c + 0.5 o2_p + pqh2_p --> h2o_p + pq_p	[11,12]
Flv2/4	sll0219, sll0217	Flavodiiron 2/4	2 h_c + 0.5 o2_u + pqh2_u --> h2o_u + pq_u	[11,12]
CBFCpp	slr0342, slr0342, sll1182, slr1185, sll1316	Cytochrome b6/f complex periplasm	Fixed to zero	[11,12]
CBFC2pp	slr0342, slr0342, sll1182, slr1185, sll1316	Cytochrome b6/f complex periplasm	Fixed to zero	[11,12]
CYO1b2pp_syn	slr1136, slr1137, slr1138	Cytochrome c oxidase	Fixed to zero	[11,12]
CYO1bpp_syn	slr1136, slr1137, slr1138	Cytochrome c oxidase	Fixed to zero	[11,12]
CYO1b2_syn	slr1136, slr1137, slr1138	Cytochrome c oxidase	Fixed to zero	[11,12]
NDH1_2p	sll0223	NAD(P)H dehydrogenase	Fixed to zero	[11,12]
NDH1_1p	sll0223	NAD(P)H dehydrogenase	Fixed to zero	[11,12]
LEUTAi	slr0032	Leucine transaminase (irreversible)	Fixed to zero	[9]
ATPM		Non-growth associated ATP maintenance	atp_c + h2o_c --> adp_c + h_c + pi_c	

Supplementary Table 2 Reactions added to iJN678_AK to create iJN678_AK_isoprene.

Name	Gene–Reaction Rule	Description	Stoichiometry	Reference
ISPs	PmIspS	Isoprene synthase from Pueraria montana	dmpp_c --> isp_c + ppi_c	[16]
ISPt		Isoprene transport via diffusion (cytoplasm to extracellular)	isp_c --> isp_e	
EX_isp_e		Isoprene exchange	isp_e -->	

Supplementary Table 3 Reactions added to iJN678 to create iJN678_AK_isobutene.

Name	Gene–Reaction Rule	Description	Stoichiometry	Reference
KICD	RnKICD	2-ketoisocaproate dioxygenase from *Rattus norvegicus*	4mop_c + o2_c --> co2_c + m_3hivac_c	[13–15]

M3K	PtM3K	mevalonate-3-kinase from *Picrophilus torridus*	atp_c + m_3hivac_c --> adp_c + m_3piv_c	[13–15]
Isobutene_spontaneous		Isobutene spontaneous	m_3piv_c --> co2_c + isb_c + pi_c	
ISBt		Isobutene transport via diffusion (cytoplasm to extracellular))	isb_c --> isb_e	
EX_isb_e		Isobutene exchange	isb_e -->	

Supplementary Table 4 Reactions added to iJN678_AK to create iJN678_AK_ethylene.

Name	Gene–Reaction Rule	Description	Stoichiometry	Reference
EFE	PsEFE	Ethylene-forming enzyme from Pseudomonas syringae	3 akg_c + arg__L_c + 3 h_c + 3 o2_c --> 1pyr5c_c + 7 co2_c + 2 ethylene_c + guanidine_c + 3 h2o_c + succ_c	[17]
GUANIDINEt		Guanidine transport via diffusion (cytoplasm to extracellular)	guanidine_c --> guanidine_e	
ETHYLENEt		Ethylene transport via diffusion (cytoplasm to extracellular)	ethylene_c --> eth_e	
EX_gua_e		Guanidine exchange	guanidine_e -->	
EX_eth_e		Ethylene exchange	eth_e -->	

Supplementary Table 5 Reactions added to iJN678_AK to create iJN678_AK_1-undecene.

Name	Gene–Reaction Rule	Description	Stoichiometry	Reference
tesA	EcTesA	Acyl-ACP thioesterase from Escherichia coli	ddcaACP_c + h2o_c --> ACP_c + dodecacid_c + h_c	[18]
UndB	PmUndB	Desaturase-like from Pseudomonas mendocina	dodecacid_c --> co2_c + h_c + und_c	[18]
UNDt		1-undecene transport via diffusion (cytoplasm to extracellular)	und_c --> und_e	
EX_und_e		1-undecene exchange	und_e -->	

Supplementary Table 6 Release of CO_2 in *Synechocystis* simulated to grow autotrophically and mixotrophically, when maximizing biomass and alkenes production. Estimated values are based on the flux-sum analysis using iJN678_2023 metabolic model of *Synechocystis*. Values are presented in units of mmol/gDW/h. Auto, autotrophic; mixo, mixotrophic.

Objective	Auto	Mixo
**biomass**	3.81	0.59
**isoprene**	4.41	0.55
**isobutene**	5.80	2.64
**ethylene**	5.27	4.52
**1-undecene**	3.99	1.17

Supplementary Table 7 Predicted flux distributions of ADP-producing reactions in *Synechocystis* simulated to grow autotrophically and mixotrophically, when maximizing biomass and alkenes production. Presented are percentage (%) and the corresponding flux (mmol/gDW/h) of reactions contributing to the generation of ADP. Zero flux indicates that the reaction carries no flux or less than 0.01 mmol/gDW/h. For the complete list of reactions, including their abbreviations, subsystem, lower and upper bounds, and stoichiometry, refer to data availability section. Auto, autotrophic; Mixo, mixotrophic. Auto, autotrophic; Mixo, mixotrophic.

		Biomass		Isoprene		Isobutene		Ethylene		1-undecene	
**trophy**	**reaction**	**flux**	**percent**	**flux**	**percent**	**flux**	**percent**	**flux**	**percent**	**flux**	**percent**
**Auto**	**ACCOAC**	0.24	1.49	0.02	0.15	0.02	0.14	0.02	0.16	1.52	10.53
	**ACGK**	0.02	0.14	0.00	0.01	0.00	0.01	0.33	2.13	0.00	0.02
	**ACKr**	0.02	0.10	0.00	0.01	0.00	0.01	0.00	0.01	0.00	0.01
	**ADCPS2**	0.00	0.00	0.00	0.00	0.00	0.00	0.00	0.00	0.00	0.00
	**ADCYRS**	0.00	0.00	0.00	0.00	0.00	0.00	0.00	0.00	0.00	0.00
	**ADK1**	0.35	2.12	0.03	0.21	0.03	0.20	0.68	4.46	0.03	0.24
	**ADSK**	0.02	0.09	0.00	0.01	0.00	0.01	0.00	0.01	0.00	0.01
	**ALAALAr**	0.00	0.01	0.00	0.00	0.00	0.00	0.00	0.00	0.00	0.00
	**ASPK**	0.07	0.43	0.01	0.04	0.01	0.04	0.01	0.05	0.01	0.05
	**BIOMASS_Ec_SynAuto**	4.40	26.94	0.44	2.63	0.44	2.55	0.44	2.87	0.44	3.04
	**CA2abcpp**	0.00	0.00	0.00	0.00	0.00	0.00	0.00	0.00	0.00	0.00
	**CBMKr**	0.05	0.29	0.00	0.03	0.00	0.03	0.33	2.15	0.00	0.03
	**CDPMEK**	0.01	0.09	0.67	4.02	0.00	0.01	0.00	0.01	0.00	0.01
	**COCHL**	0.00	0.00	0.00	0.00	0.00	0.00	0.00	0.00	0.00	0.00
	**CTPS1**	0.01	0.07	0.00	0.01	0.00	0.01	0.00	0.01	0.00	0.01
	**CUabcpp**	0.00	0.00	0.00	0.00	0.00	0.00	0.00	0.00	0.00	0.00
	**CYTK1**	0.02	0.10	0.67	4.02	0.00	0.01	0.00	0.01	0.00	0.01
	**Cobalt2abcppI**	0.00	0.00	0.00	0.00	0.00	0.00	0.00	0.00	0.00	0.00
	**DHFS**	0.00	0.00	0.00	0.00	0.00	0.00	0.00	0.00	0.00	0.00
	**DPCOAK**	0.00	0.00	0.00	0.00	0.00	0.00	0.00	0.00	0.00	0.00
	**DTMPK**	0.00	0.01	0.00	0.00	0.00	0.00	0.00	0.00	0.00	0.00
	**FE2abcpp**	0.00	0.00	0.00	0.00	0.00	0.00	0.00	0.00	0.00	0.00
	**FE3abcpp**	0.00	0.00	0.00	0.00	0.00	0.00	0.00	0.00	0.00	0.00
	**FTHFLi**	0.00	0.00	0.00	0.00	0.00	0.00	0.00	0.00	0.00	0.00
	**GK1**	0.01	0.08	0.00	0.01	0.00	0.01	0.00	0.01	0.00	0.01

	**GLCS1**	0.02	0.11	0.00	0.01	0.00	0.01	0.00	0.01	0.00	0.01
	**GLNS**	0.11	0.68	0.01	0.07	0.01	0.06	0.38	2.51	0.02	0.10
	**GLU5K**	0.02	0.12	0.00	0.01	0.00	0.01	0.00	0.00	0.00	0.01
	**GLUCYS**	0.00	0.00	0.00	0.00	0.00	0.00	0.00	0.00	0.00	0.00
	**GTHS**	0.00	0.00	0.00	0.00	0.00	0.00	0.00	0.00	0.00	0.00
	**HSK**	0.04	0.27	0.00	0.03	0.00	0.03	0.00	0.03	0.00	0.03
	**LTHRK**	0.00	0.00	0.00	0.00	0.00	0.00	0.00	0.00	0.00	0.00
	**MG2uabcpp**	0.00	0.01	0.00	0.00	0.00	0.00	0.00	0.00	0.00	0.00
	**MOBDabcpp**	0.00	0.00	0.00	0.00	0.00	0.00	0.00	0.00	0.00	0.00
	**MPML**	0.00	0.01	0.00	0.00	0.00	0.00	0.00	0.00	0.00	0.00
	**NADK**	0.00	0.00	0.00	0.00	0.00	0.00	0.00	0.00	0.00	0.00
	**NDPK3**	0.01	0.09	0.00	0.00	0.00	0.00	0.00	0.01	0.00	0.00
	**NDPK4**	0.00	0.01	0.00	0.00	0.00	0.00	0.00	0.00	0.00	0.00
	**NDPK5**	0.00	0.01	0.00	0.00	0.00	0.00	0.00	0.00	0.00	0.00
	**NDPK7**	0.00	0.01	0.00	0.00	0.00	0.00	0.00	0.00	0.00	0.00
	**NDPK8**	0.00	0.01	0.00	0.00	0.00	0.00	0.00	0.00	0.00	0.00
	**NO3abcpp**	0.74	4.55	0.07	0.44	0.07	0.43	1.05	6.84	0.07	0.51
	**PGK**	6.37	39.04	8.02	47.97	10.12	58.80	7.78	50.76	7.84	54.14
	**PIuabcpp**	0.06	0.35	0.01	0.03	0.01	0.03	0.01	0.04	0.01	0.04
	**PNTK**	0.00	0.00	0.00	0.00	0.00	0.00	0.00	0.00	0.00	0.00
	**PRAGSr**	0.03	0.16	0.00	0.02	0.00	0.02	0.00	0.02	0.00	0.02
	**PRAIS**	0.03	0.16	0.00	0.02	0.00	0.02	0.00	0.02	0.00	0.02
	**PRASCSi**	0.03	0.16	0.00	0.02	0.00	0.02	0.00	0.02	0.00	0.02
	**PRFGS**	0.03	0.16	0.00	0.02	0.00	0.02	0.00	0.02	0.00	0.02
	**PRUK**	3.50	21.43	4.38	26.18	5.77	33.52	4.25	27.70	3.95	27.29
	**R05224_1**	0.00	0.00	0.00	0.00	0.00	0.00	0.00	0.00	0.00	0.00
	**RBFK**	0.00	0.00	0.00	0.00	0.00	0.00	0.00	0.00	0.00	0.00
	**SHKK**	0.03	0.20	0.00	0.02	0.00	0.02	0.00	0.02	0.00	0.02
	**SUCOAS**	0.02	0.11	0.00	0.01	0.00	0.01	0.00	0.01	0.00	0.01
	**SULabcpp**	0.02	0.10	0.00	0.01	0.00	0.01	0.00	0.01	0.00	0.01
	**UAAGDS**	0.00	0.01	0.00	0.00	0.00	0.00	0.00	0.00	0.00	0.00
	**UAMAGS**	0.00	0.01	0.00	0.00	0.00	0.00	0.00	0.00	0.00	0.00
	**UAMAS**	0.00	0.01	0.00	0.00	0.00	0.00	0.00	0.00	0.00	0.00
	**UGMDDS**	0.00	0.01	0.00	0.00	0.00	0.00	0.00	0.00	0.00	0.00
	**UMPK**	0.03	0.18	0.00	0.02	0.00	0.02	0.00	0.02	0.00	0.02
	**ZNabcpp**	0.00	0.00	0.00	0.00	0.00	0.00	0.00	0.00	0.00	0.00
	**BCT1_syn**	0.00	0.00	2.34	13.98	0.00	0.00	0.00	0.02	0.54	3.72
	**GLYK**	0.00	0.00	0.00	0.00	0.00	0.01	0.00	0.01	0.00	0.01
	**M3K**	0.00	0.00	0.00	0.00	0.68	3.94	0.00	0.00	0.00	0.00
	**M3K**	0.00	0.00	0.00	0.00	0.51	4.40	0.00	0.00	0.00	0.00
	**NDPK1**	0.00	0.00	0.00	0.00	0.00	0.00	0.00	0.02	0.00	0.00
	**NDPK2**	0.00	0.00	0.00	0.00	0.00	0.00	0.00	0.03	0.00	0.00
**Mixo**	**ACCOAC**	0.17	1.55	0.02	0.16	0.02	0.14	0.02	0.12	0.95	8.82
	**ACGK**	0.02	0.14	0.00	0.01	0.00	0.01	0.41	3.02	0.00	0.01
	**ACKr**	0.01	0.10	0.00	0.01	0.00	0.01	0.00	0.01	0.00	0.01
	**ADCPS2**	0.00	0.00	0.00	0.00	0.00	0.00	0.00	0.00	0.00	0.00

	**ADCYRS**	0.00	0.00	0.00	0.00	0.00	0.00	0.00	0.00	0.00	0.00
	**ADK1**	0.24	2.20	0.02	0.22	0.02	0.20	2.01	14.77	0.02	0.22
	**ADSK**	0.01	0.10	0.00	0.01	0.00	0.01	0.00	0.01	0.00	0.01
	**ALAALAr**	0.00	0.01	0.00	0.00	0.00	0.00	0.00	0.00	0.00	0.00
	**ASPK**	0.05	0.45	0.00	0.05	0.00	0.04	0.00	0.04	0.00	0.04
	**BIOMASS_Ec_SynMixo**	2.16	20.14	0.22	2.05	0.22	1.85	0.22	1.58	0.22	2.00
	**CA2abcpp**	0.00	0.00	0.00	0.00	0.00	0.00	0.00	0.00	0.00	0.00
	**CBPS**	0.07	0.61	0.01	0.06	0.01	0.06	0.00	0.00	0.01	0.06
	**CDPMEK**	0.01	0.09	0.41	3.92	0.00	0.01	0.00	0.01	0.00	0.01
	**COCHL**	0.00	0.00	0.00	0.00	0.00	0.00	0.00	0.00	0.00	0.00
	**CTPS1**	0.01	0.08	0.00	0.01	0.00	0.01	0.00	0.01	0.00	0.01
	**CUabcpp**	0.00	0.00	0.00	0.00	0.00	0.00	0.00	0.00	0.00	0.00
	**CYPHYS**	6.08	56.82	0.00	0.00	5.27	45.18	0.00	0.00	8.53	79.18
	**CYTK1**	0.01	0.10	0.41	3.92	0.00	0.01	0.00	0.01	0.00	0.01
	**Cobalt2abcppI**	0.00	0.00	0.00	0.00	0.00	0.00	0.00	0.00	0.00	0.00
	**DHFS**	0.00	0.00	0.00	0.00	0.00	0.00	0.00	0.00	0.00	0.00
	**DPCOAK**	0.00	0.00	0.00	0.00	0.00	0.00	0.00	0.00	0.00	0.00
	**DTMPK**	0.00	0.01	0.00	0.00	0.00	0.00	0.00	0.00	0.00	0.00
	**FE2abcpp**	0.00	0.00	0.00	0.00	0.00	0.00	0.00	0.00	0.00	0.00
	**FE3abcpp**	0.00	0.00	0.00	0.00	0.00	0.00	0.00	0.00	0.00	0.00
	**FTHFLi**	0.00	0.00	0.00	0.00	0.00	0.01	0.00	0.00	0.00	0.00
	**GK1**	0.01	0.08	0.00	0.01	0.00	0.01	0.00	0.01	0.00	0.01
	**GLNS**	0.52	4.85	0.05	0.47	0.05	0.43	1.09	8.00	0.05	0.48
	**GLU5K**	0.01	0.12	0.00	0.01	0.00	0.01	0.00	0.00	0.00	0.01
	**GLUCYS**	0.00	0.00	0.00	0.00	0.00	0.00	0.00	0.00	0.00	0.00
	**GLYCK**	0.00	0.00	0.00	0.00	0.00	0.00	0.00	0.00	0.00	0.01
	**GTHS**	0.00	0.00	0.00	0.00	0.00	0.00	0.00	0.00	0.00	0.00
	**HEX1**	0.38	3.55	0.38	3.62	0.38	3.26	0.38	2.79	0.38	3.53
	**HSK**	0.03	0.28	0.00	0.03	0.00	0.03	0.00	0.02	0.00	0.03
	**LTHRK**	0.00	0.00	0.00	0.00	0.00	0.00	0.00	0.00	0.00	0.00
	**MG2uabcpp**	0.00	0.02	0.00	0.00	0.00	0.00	0.00	0.00	0.00	0.00
	**MOBDabcpp**	0.00	0.00	0.00	0.00	0.00	0.00	0.00	0.00	0.00	0.00
	**MPML**	0.00	0.01	0.00	0.00	0.00	0.00	0.00	0.00	0.00	0.00
	**NADK**	0.00	0.00	0.00	0.00	0.00	0.00	0.00	0.00	0.00	0.00
	**NDPK1**	0.02	0.20	0.00	0.02	0.00	0.00	0.00	0.02	0.00	0.00
	**NDPK2**	0.03	0.30	0.00	0.03	0.00	0.00	0.00	0.02	0.00	0.00
	**NDPK3**	0.01	0.09	0.07	0.66	0.00	0.00	0.00	0.01	0.00	0.00
	**NDPK4**	0.00	0.01	0.00	0.00	0.00	0.00	0.00	0.00	0.00	0.00
	**NDPK5**	0.00	0.01	0.00	0.00	0.00	0.00	0.00	0.00	0.00	0.00
	**NDPK7**	0.00	0.01	0.00	0.00	0.00	0.00	0.00	0.00	0.00	0.00
	**NDPK8**	0.00	0.01	0.00	0.00	0.00	0.00	0.00	0.00	0.00	0.00
	**NO3abcpp**	0.51	4.73	0.05	0.48	0.05	0.43	1.28	9.41	0.05	0.47
	**PIuabcpp**	0.04	0.36	0.00	0.04	0.00	0.03	0.00	0.03	0.00	0.04
	**PNTK**	0.00	0.00	0.00	0.00	0.00	0.00	0.00	0.00	0.00	0.00
	**PRAGSr**	0.02	0.17	0.00	0.02	0.00	0.02	0.00	0.01	0.00	0.02
	**PRAIS**	0.02	0.17	0.00	0.02	0.00	0.02	0.00	0.01	0.00	0.02

	**PRASCSi**	0.02	0.17	0.00	0.02	0.00	0.02	0.00	0.01	0.00	0.02
	**PRFGS**	0.02	0.17	0.00	0.02	0.00	0.02	0.00	0.01	0.00	0.02
	**PRUK**	0.17	1.56	0.41	3.94	2.06	17.63	2.88	21.11	0.19	1.76
	**R05224_1**	0.00	0.00	0.00	0.00	0.00	0.00	0.00	0.00	0.00	0.00
	**RBFK**	0.00	0.00	0.00	0.00	0.00	0.00	0.00	0.00	0.00	0.00
	**SHKK**	0.02	0.21	0.00	0.02	0.00	0.02	0.00	0.02	0.00	0.02
	**SUCOAS**	0.01	0.11	0.00	0.01	0.00	0.01	0.00	0.01	0.00	0.01
	**SULabcpp**	0.01	0.10	0.00	0.01	0.00	0.01	0.00	0.01	0.00	0.01
	**UAAGDS**	0.00	0.01	0.00	0.00	0.00	0.00	0.00	0.00	0.00	0.00
	**UAMAGS**	0.00	0.01	0.00	0.00	0.00	0.00	0.00	0.00	0.00	0.00
	**UAMAS**	0.00	0.01	0.00	0.00	0.00	0.00	0.00	0.00	0.00	0.00
	**UGMDDS**	0.00	0.01	0.00	0.00	0.00	0.00	0.00	0.00	0.00	0.00
	**UMPK**	0.02	0.19	0.00	0.02	0.00	0.02	0.00	0.01	0.00	0.02
	**ZNabcpp**	0.00	0.00	0.00	0.00	0.00	0.00	0.00	0.00	0.00	0.00
	**GLYK**	0.00	0.00	0.00	0.00	0.00	0.00	0.00	0.01	0.00	0.00
	**NGAM**	0.00	0.00	7.96	75.80	0.00	0.00	0.00	0.00	0.00	0.00
	**PFK**	0.00	0.00	0.00	0.01	0.00	0.00	0.00	0.00	0.00	0.00
	**PGK**	0.00	0.00	0.45	4.31	3.04	26.10	4.89	35.87	0.34	3.13
	**CBMKr**	0.00	0.00	0.00	0.00	0.00	0.00	0.41	3.04	0.00	0.00

Supplementary Table 8 Predicted flux distributions of ADP-consuming reactions in *Synechocystis* simulated to grow autotrophically and mixotrophically, when maximizing biomass and alkenes production. Presented are percentage (%) and the corresponding flux (mmol/gDW/h) of reactions contributing to the consumption of ADP. Zero flux indicates that the reaction carries no flux or less than 0.01 mmol/gDW/h. For the complete list of reactions, including their abbreviations, subsystem, lower and upper bounds, and stoichiometry, refer to data availability section. Auto, autotrophic; Mixo, mixotrophic.

		Biomass		Isoprene		Isobutene		Ethylene		1-undecene	
**trophy**	**reaction**	**flux**	**percent**	**flux**	**percent**	**flux**	**percent**	**flux**	**percent**	**flux**	**percent**
**Auto**	**ATPSu**	-16.04	98.27	-16.19	96.84	-15.84	91.97	-15.04	98.09	-14.46	99.95
	**PYK**	-0.15	0.91	-0.01	0.08	-1.37	7.95	0.00	0.00	-0.01	0.04
	**ATPS4rpp_1**	-0.13	0.80	-0.51	3.07	-0.01	0.07	-0.29	1.90	0.00	0.00
	**URIDK2r**	0.00	0.01	0.00	0.00	0.00	0.00	0.00	0.00	0.00	0.00
	**RNDR1**	0.00	0.01	0.00	0.00	0.00	0.00	0.00	0.00	0.00	0.00
	**PPK2**	0.00	0.00	0.00	0.00	0.00	0.00	0.00	0.00	0.00	0.00
	**NDPK6**	0.00	0.00	0.00	0.00	0.00	0.00	0.00	0.00	0.00	0.00
**Mixo**	**ATPSu**	-10.70	99.97	-10.50	100.00	-11.10	95.25	-13.44	98.65	-10.76	99.96
	**URIDK2r**	0.00	0.01	0.00	0.00	0.00	0.00	0.00	0.00	0.00	0.00
	**RNDR1**	0.00	0.01	0.00	0.00	0.00	0.00	0.00	0.00	0.00	0.00
	**PPK2**	0.00	0.00	0.00	0.00	0.00	0.00	0.00	0.00	0.00	0.00
	**PYK**	0.00	0.00	0.00	0.00	-0.55	4.75	0.00	0.00	0.00	0.04
	**ATPS4rpp_1**	0.00	0.00	0.00	0.00	0.00	0.00	-0.18	1.35	0.00	0.00

Supplementary Table 9 Predicted flux distributions of AMP-producing reactions in *Synechocystis* simulated to grow autotrophically and mixotrophically, when maximizing biomass and alkenes production. Presented are percentage (%) and the corresponding flux (mmol/gDW/h) of reactions contributing to the generation of AMP. Zero flux indicates that the reaction carries no flux or less than 0.01 mmol/gDW/h. For the complete list of reactions, including their abbreviations, subsystem, lower and upper bounds, and stoichiometry, refer to data availability section. Auto, autotrophic; Mixo, mixotrophic.

		Biomass		Isoprene		Isobutene		Ethylene		1-undecene	
**trophy**	**reaction**	**flux**	**percent**	**flux**	**percent**	**flux**	**percent**	**flux**	**percent**	**flux**	**percent**
**Auto**	**ADPT**	0.00	1.52	0.00	1.52	0.00	1.52	0.00	0.08	0.00	1.52
	**ADSL1r**	0.02	12.01	0.00	12.01	0.00	12.01	0.00	0.61	0.00	12.01
	**ARGSS**	0.02	12.98	0.00	12.98	0.00	12.98	0.33	95.61	0.00	12.98
	**ASNS1**	0.02	8.90	0.00	8.90	0.00	8.90	0.00	0.45	0.00	8.90
	**BPNT**	0.02	8.87	0.00	8.87	0.00	8.87	0.00	0.45	0.00	8.87
	**GLUTRS**	0.01	8.43	0.00	8.43	0.00	8.43	0.00	0.43	0.00	8.43
	**GMPS**	0.01	7.54	0.00	7.54	0.00	7.54	0.00	0.38	0.00	7.54
	**HPPK**	0.00	0.04	0.00	0.04	0.00	0.04	0.00	0.00	0.00	0.04
	**NADS2**	0.00	0.11	0.00	0.11	0.00	0.11	0.00	0.01	0.00	0.11
	**PANTS**	0.00	0.03	0.00	0.03	0.00	0.03	0.00	0.00	0.00	0.03
	**PRPPS**	0.07	39.47	0.01	39.47	0.01	39.47	0.01	1.99	0.01	39.47
	**SUCBZL**	0.00	0.08	0.00	0.08	0.00	0.08	0.00	0.00	0.00	0.08
**Mixo**	**ADPT**	0.00	1.52	0.00	1.52	0.00	1.52	0.00	0.02	0.00	1.52
	**ADSL1r**	0.01	12.01	0.00	12.01	0.00	12.01	0.00	0.14	0.00	12.01
	**ARGSS**	0.02	12.98	0.00	12.98	0.00	12.98	0.41	40.95	0.00	12.98
	**ASNS1**	0.01	8.90	0.00	8.90	0.00	8.90	0.00	0.10	0.00	8.90
	**BPNT**	0.01	8.87	0.00	8.87	0.00	8.87	0.00	0.10	0.00	8.87
	**GLUTRS**	0.01	8.43	0.00	8.43	0.00	8.43	0.00	0.10	0.00	8.43
	**GMPS2**	0.01	7.54	0.00	0.00	0.00	7.54	0.00	0.00	0.00	7.54
	**HPPK**	0.00	0.04	0.00	0.04	0.00	0.04	0.00	0.00	0.00	0.04
	**NADS2**	0.00	0.11	0.00	0.11	0.00	0.11	0.00	0.00	0.00	0.11
	**PANTS**	0.00	0.03	0.00	0.03	0.00	0.03	0.00	0.00	0.00	0.03
	**PRPPS**	0.05	39.47	0.00	39.47	0.00	39.47	0.00	0.46	0.00	39.47
	**SUCBZL**	0.00	0.08	0.00	0.08	0.00	0.08	0.00	0.00	0.00	0.08
	**GMPS**	0.00	0.00	0.00	7.54	0.00	0.00	0.00	0.09	0.00	0.00
	**PPS**	0.00	0.00	0.00	0.00	0.00	0.00	0.58	58.03	0.00	0.00

Supplementary Table 10 Predicted flux distributions of AMP-consuming reactions in *Synechocystis* simulated to grow autotrophically and mixotrophically, when maximizing biomass and alkenes production. Presented are percentage (%) and the corresponding flux (mmol/gDW/h) of reactions contributing to the consumption of AMP. Zero flux indicates that the reaction carries no flux or less than 0.01 mmol/gDW/h. For the complete list of reactions, including their abbreviations, subsystem, lower and upper bounds, and stoichiometry, refer to data availability section. Auto, autotrophic; Mixo, mixotrophic.

		Biomass		Isoprene		Isobutene		Ethylene		1-undecene	
**trophy**	**reaction**	**flux**	**percent**	**flux**	**percent**	**flux**	**percent**	**flux**	**percent**	**flux**	**percent**
**Auto**	**ADK1**	-0.17	100.00	-0.02	100.00	-0.02	100.00	-0.34	100.00	-0.02	100.00
**Mixo**	**ADK1**	-0.12	100.00	-0.01	100.00	-0.01	100.00	-1.01	100.00	-0.01	100.00

Supplementary Table 11 Predicted flux distributions of Pi-producing reactions in *Synechocystis* simulated to grow autotrophically and mixotrophically, when maximizing biomass and alkenes production. Presented are percentage (%) and the corresponding flux (mmol/gDW/h) of reactions contributing to the generation of Pi. Zero flux indicates that the reaction carries no flux or less than 0.01 mmol/gDW/h. For the complete list of reactions, including their abbreviations, subsystem, lower and upper bounds, and stoichiometry, refer to data availability section. Auto, autotrophic; Mixo, mixotrophic.

		Biomass		Isoprene		Isobutene		Ethylene		1-undecene	
**trophy**	**reaction**	**flux**	**percent**	**flux**	**percent**	**flux**	**percent**	**flux**	**percent**	**flux**	**percent**
**Auto**	**ACCOAC**	0.24	1.46	0.02	0.14	0.02	0.15	0.02	0.15	1.52	9.35
	**ADCPS2**	0.00	0.00	0.00	0.00	0.00	0.00	0.00	0.00	0.00	0.00
	**ADCYRS**	0.00	0.00	0.00	0.00	0.00	0.00	0.00	0.00	0.00	0.00
	**ADSS**	0.02	0.12	0.00	0.01	0.00	0.01	0.00	0.01	0.00	0.01
	**AGPR**	0.02	0.14	0.00	0.01	0.00	0.01	0.33	2.00	0.00	0.01
	**ALAALAr**	0.00	0.01	0.00	0.00	0.00	0.00	0.00	0.00	0.00	0.00
	**ASAD**	0.07	0.43	0.01	0.04	0.01	0.04	0.01	0.04	0.01	0.04
	**ASPCT**	0.03	0.15	0.00	0.02	0.00	0.02	0.00	0.02	0.00	0.02
	**BIOMASS_Ec_SynAuto**	4.40	26.49	0.44	2.63	0.44	2.65	0.44	2.69	0.44	2.70
	**BPNT**	0.02	0.09	0.00	0.01	0.00	0.01	0.00	0.01	0.00	0.01
	**CA2abcpp**	0.00	0.00	0.00	0.00	0.00	0.00	0.00	0.00	0.00	0.00
	**CHORS**	0.03	0.20	0.00	0.02	0.00	0.02	0.00	0.02	0.00	0.02
	**COCHL**	0.00	0.00	0.00	0.00	0.00	0.00	0.00	0.00	0.00	0.00
	**CTPS1**	0.01	0.07	0.00	0.01	0.00	0.01	0.00	0.01	0.00	0.01
	**CUabcpp**	0.00	0.00	0.00	0.00	0.00	0.00	0.00	0.00	0.00	0.00
	**Cobalt2abcppI**	0.00	0.00	0.00	0.00	0.00	0.00	0.00	0.00	0.00	0.00
	**DDPA**	0.03	0.20	0.00	0.02	0.00	0.02	0.00	0.02	0.00	0.02
	**DHFS**	0.00	0.00	0.00	0.00	0.00	0.00	0.00	0.00	0.00	0.00
	**DHQS**	0.03	0.20	0.00	0.02	0.00	0.02	0.00	0.02	0.00	0.02
	**DNMPPA**	0.00	0.00	0.00	0.00	0.00	0.00	0.00	0.00	0.00	0.00
	**FE2abcpp**	0.00	0.00	0.00	0.00	0.00	0.00	0.00	0.00	0.00	0.00
	**FE3abcpp**	0.00	0.00	0.00	0.00	0.00	0.00	0.00	0.00	0.00	0.00
	**FTHFLi**	0.00	0.00	0.00	0.00	0.00	0.00	0.00	0.00	0.00	0.00
	**G5SD**	0.02	0.12	0.00	0.01	0.00	0.01	0.00	0.00	0.00	0.01
	**GAPDi_nadp**	6.37	38.39	8.02	47.88	10.12	61.10	7.78	47.60	7.84	48.05

	GLNS	0.11	0.67	0.01	0.07	0.01	0.07	0.38	2.35	0.02	0.09
	GLUCYS	0.00	0.00	0.00	0.00	0.00	0.00	0.00	0.00	0.00	0.00
	GTHS	0.00	0.00	0.00	0.00	0.00	0.00	0.00	0.00	0.00	0.00
	HISTP	0.01	0.04	0.00	0.00	0.00	0.00	0.00	0.00	0.00	0.00
	METAT	0.00	0.02	0.00	0.00	0.00	0.00	0.00	0.00	0.00	0.00
	MG2uabcpp	0.00	0.01	0.00	0.00	0.00	0.00	0.00	0.00	0.00	0.00
	MOBDabcpp	0.00	0.00	0.00	0.00	0.00	0.00	0.00	0.00	0.00	0.00
	MPML	0.00	0.01	0.00	0.00	0.00	0.00	0.00	0.00	0.00	0.00
	NO3abcpp	0.74	4.48	0.07	0.44	0.07	0.45	1.05	6.41	0.07	0.46
	OCBT	0.02	0.14	0.00	0.01	0.00	0.01	0.33	2.00	0.00	0.01
	PAPA160	0.01	0.04	0.00	0.00	0.00	0.00	0.00	0.00	0.00	0.00
	PAPA161	0.00	0.00	0.00	0.00	0.00	0.00	0.00	0.00	0.00	0.00
	PAPA180	0.00	0.00	0.00	0.00	0.00	0.00	0.00	0.00	0.00	0.00
	PAPA181	0.00	0.00	0.00	0.00	0.00	0.00	0.00	0.00	0.00	0.00
	PAPA181_9	0.00	0.00	0.00	0.00	0.00	0.00	0.00	0.00	0.00	0.00
	PAPA182_9_12	0.00	0.01	0.00	0.00	0.00	0.00	0.00	0.00	0.00	0.00
	PAPA183_6_9_12	0.00	0.01	0.00	0.00	0.00	0.00	0.00	0.00	0.00	0.00
	PAPA183_9_12_15	0.00	0.00	0.00	0.00	0.00	0.00	0.00	0.00	0.00	0.00
	PAPA184_6_9_12_15	0.00	0.00	0.00	0.00	0.00	0.00	0.00	0.00	0.00	0.00
	PGPP160	0.00	0.00	0.00	0.00	0.00	0.00	0.00	0.00	0.00	0.00
	PGPP161	0.00	0.00	0.00	0.00	0.00	0.00	0.00	0.00	0.00	0.00
	PGPP180	0.00	0.00	0.00	0.00	0.00	0.00	0.00	0.00	0.00	0.00
	PGPP181	0.00	0.00	0.00	0.00	0.00	0.00	0.00	0.00	0.00	0.00
	PGPP181_9	0.00	0.00	0.00	0.00	0.00	0.00	0.00	0.00	0.00	0.00
	PGPP182_9_12	0.00	0.00	0.00	0.00	0.00	0.00	0.00	0.00	0.00	0.00
	PGPP183_9_12_15	0.00	0.00	0.00	0.00	0.00	0.00	0.00	0.00	0.00	0.00
	PIuabcpp	0.11	0.69	0.01	0.07	0.01	0.07	0.01	0.07	0.01	0.07
	PMDPHT	0.00	0.00	0.00	0.00	0.00	0.00	0.00	0.00	0.00	0.00
	PPA	0.57	3.41	2.74	16.37	0.06	0.34	0.71	4.32	0.06	0.35
	PPC	0.23	1.41	0.02	0.14	0.02	0.14	0.70	4.26	0.03	0.19
	PPNCL	0.00	0.00	0.00	0.00	0.00	0.00	0.00	0.00	0.00	0.00
	PRAGSr	0.03	0.16	0.00	0.02	0.00	0.02	0.00	0.02	0.00	0.02
	PRAIS	0.03	0.16	0.00	0.02	0.00	0.02	0.00	0.02	0.00	0.02
	PRASCSi	0.03	0.16	0.00	0.02	0.00	0.02	0.00	0.02	0.00	0.02
	PRFGS	0.03	0.16	0.00	0.02	0.00	0.02	0.00	0.02	0.00	0.02
	PSCVT	0.03	0.20	0.00	0.02	0.00	0.02	0.00	0.02	0.00	0.02
	PSP	0.10	0.57	0.01	0.06	0.01	0.07	0.01	0.06	0.01	0.06
	PTAr	0.44	2.67	0.04	0.26	0.72	4.36	1.02	6.23	1.84	11.31
	QULNS	0.00	0.00	0.00	0.00	0.00	0.00	0.00	0.00	0.00	0.00
	RBFSa	0.00	0.00	0.00	0.00	0.00	0.00	0.00	0.00	0.00	0.00
	SBP	2.71	16.34	1.47	8.80	2.17	13.07	1.76	10.74	1.93	11.85
	SUCOAS	0.02	0.11	0.00	0.01	0.00	0.01	0.00	0.01	0.00	0.01
	SULabcpp	0.02	0.09	0.00	0.01	0.00	0.01	0.00	0.01	0.00	0.01
	THRS	0.04	0.27	0.00	0.03	0.00	0.03	0.00	0.03	0.00	0.03
	UAAGDS	0.00	0.01	0.00	0.00	0.00	0.00	0.00	0.00	0.00	0.00
	UAGCVT	0.00	0.01	0.00	0.00	0.00	0.00	0.00	0.00	0.00	0.00

	UAMAGS	0.00	0.01	0.00	0.00	0.00	0.00	0.00	0.00	0.00	0.00
	UAMAS	0.00	0.01	0.00	0.00	0.00	0.00	0.00	0.00	0.00	0.00
	UDCPDP	0.00	0.01	0.00	0.00	0.00	0.00	0.00	0.00	0.00	0.00
	UGMDDS	0.00	0.01	0.00	0.00	0.00	0.00	0.00	0.00	0.00	0.00
	ZNabcpp	0.00	0.00	0.00	0.00	0.00	0.00	0.00	0.00	0.00	0.00
	BCT1_syn	0.00	0.00	2.34	13.95	0.00	0.00	0.00	0.02	0.54	3.30
	FBP	0.00	0.00	1.48	8.85	2.17	13.11	1.76	10.79	1.94	11.89
	Isobutene_spontaneous	0.00	0.00	0.00	0.00	0.68	4.09	0.00	0.00	0.00	0.00
**Mixo**	ACCOAC	0.17	1.50	0.02	0.16	0.02	0.14	0.02	0.11	0.95	7.97
	ADCPS2	0.00	0.00	0.00	0.00	0.00	0.00	0.00	0.00	0.00	0.00
	ADCYRS	0.00	0.00	0.00	0.00	0.00	0.00	0.00	0.00	0.00	0.00
	ADSS	0.01	0.13	0.00	0.01	0.00	0.01	0.00	0.01	0.00	0.01
	AGPR	0.02	0.14	0.00	0.01	0.00	0.01	0.41	2.77	0.00	0.01
	ALAALAr	0.00	0.01	0.00	0.00	0.00	0.00	0.00	0.00	0.00	0.00
	ASAD	0.05	0.44	0.00	0.05	0.00	0.04	0.00	0.03	0.00	0.04
	ASPCT	0.02	0.16	0.00	0.02	0.00	0.02	0.00	0.01	0.00	0.01
	BIOMASS_Ec_SynMixo	2.15	19.61	0.22	2.05	0.22	1.85	0.22	1.45	0.22	1.81
	BPNT	0.01	0.09	0.00	0.01	0.00	0.01	0.00	0.01	0.00	0.01
	CA2abcpp	0.00	0.00	0.00	0.00	0.00	0.00	0.00	0.00	0.00	0.00
	CBPS	0.03	0.30	0.00	0.03	0.00	0.03	0.00	0.00	0.00	0.03
	CHORS	0.02	0.20	0.00	0.02	0.00	0.02	0.00	0.01	0.00	0.02
	COCHL	0.00	0.00	0.00	0.00	0.00	0.00	0.00	0.00	0.00	0.00
	CTPS1	0.01	0.08	0.00	0.01	0.00	0.01	0.00	0.01	0.00	0.01
	CUabcpp	0.00	0.00	0.00	0.00	0.00	0.00	0.00	0.00	0.00	0.00
	CYPHYS	6.08	55.32	0.00	0.00	5.27	45.22	0.00	0.00	8.53	71.58
	Cobalt2abcppI	0.00	0.00	0.00	0.00	0.00	0.00	0.00	0.00	0.00	0.00
	DDPA	0.02	0.20	0.00	0.02	0.00	0.02	0.00	0.01	0.00	0.02
	DHFS	0.00	0.00	0.00	0.00	0.00	0.00	0.00	0.00	0.00	0.00
	DHQS	0.02	0.20	0.00	0.02	0.00	0.02	0.00	0.01	0.00	0.02
	DNMPPA	0.00	0.00	0.00	0.00	0.00	0.00	0.00	0.00	0.00	0.00
	FE2abcpp	0.00	0.00	0.00	0.00	0.00	0.00	0.00	0.00	0.00	0.00
	FE3abcpp	0.00	0.00	0.00	0.00	0.00	0.00	0.00	0.00	0.00	0.00
	FTHFLi	0.00	0.00	0.00	0.00	0.00	0.01	0.00	0.00	0.00	0.00
	G5SD	0.01	0.12	0.00	0.01	0.00	0.01	0.00	0.00	0.00	0.01
	GLNS	0.52	4.73	0.05	0.47	0.05	0.43	1.09	7.32	0.05	0.43
	GLUCYS	0.00	0.00	0.00	0.00	0.00	0.00	0.00	0.00	0.00	0.00
	GTHS	0.00	0.00	0.00	0.00	0.00	0.00	0.00	0.00	0.00	0.00
	HISTP	0.00	0.04	0.00	0.00	0.00	0.00	0.00	0.00	0.00	0.00
	METAT	0.00	0.02	0.00	0.00	0.00	0.00	0.00	0.00	0.00	0.00
	MG2uabcpp	0.00	0.01	0.00	0.00	0.00	0.00	0.00	0.00	0.00	0.00
	MOBDabcpp	0.00	0.00	0.00	0.00	0.00	0.00	0.00	0.00	0.00	0.00
	MPML	0.00	0.01	0.00	0.00	0.00	0.00	0.00	0.00	0.00	0.00
	NO3abcpp	0.51	4.61	0.05	0.48	0.05	0.43	1.28	8.61	0.05	0.42
	OCBT	0.02	0.14	0.00	0.01	0.00	0.01	0.41	2.77	0.00	0.01
	PAPA160	0.00	0.04	0.00	0.00	0.00	0.00	0.00	0.00	0.00	0.00
	PAPA161	0.00	0.00	0.00	0.00	0.00	0.00	0.00	0.00	0.00	0.00

	PAPA180	0.00	0.00	0.00	0.00	0.00	0.00	0.00	0.00	0.00	0.00
	PAPA181	0.00	0.00	0.00	0.00	0.00	0.00	0.00	0.00	0.00	0.00
	PAPA181_9	0.00	0.00	0.00	0.00	0.00	0.00	0.00	0.00	0.00	0.00
	PAPA182_9_12	0.00	0.01	0.00	0.00	0.00	0.00	0.00	0.00	0.00	0.00
	PAPA183_6_9_12	0.00	0.01	0.00	0.00	0.00	0.00	0.00	0.00	0.00	0.00
	PAPA183_9_12_15	0.00	0.00	0.00	0.00	0.00	0.00	0.00	0.00	0.00	0.00
	PAPA184_6_9_12_15	0.00	0.00	0.00	0.00	0.00	0.00	0.00	0.00	0.00	0.00
	PGPP160	0.00	0.00	0.00	0.00	0.00	0.00	0.00	0.00	0.00	0.00
	PGPP161	0.00	0.00	0.00	0.00	0.00	0.00	0.00	0.00	0.00	0.00
	PGPP180	0.00	0.00	0.00	0.00	0.00	0.00	0.00	0.00	0.00	0.00
	PGPP181	0.00	0.00	0.00	0.00	0.00	0.00	0.00	0.00	0.00	0.00
	PGPP181_9	0.00	0.00	0.00	0.00	0.00	0.00	0.00	0.00	0.00	0.00
	PGPP182_9_12	0.00	0.00	0.00	0.00	0.00	0.00	0.00	0.00	0.00	0.00
	PGPP183_9_12_15	0.00	0.00	0.00	0.00	0.00	0.00	0.00	0.00	0.00	0.00
	PIuabcpp	0.08	0.71	0.01	0.07	0.01	0.07	0.01	0.05	0.01	0.07
	PMDPHT	0.00	0.00	0.00	0.00	0.00	0.00	0.00	0.00	0.00	0.00
	PPA	0.36	3.29	1.68	15.93	0.04	0.31	0.86	5.76	0.04	0.30
	PPC	0.21	1.88	0.02	0.20	0.50	4.28	1.21	8.15	0.02	0.17
	PPNCL	0.00	0.00	0.00	0.00	0.00	0.00	0.00	0.00	0.00	0.00
	PRAGSr	0.02	0.17	0.00	0.02	0.00	0.02	0.00	0.01	0.00	0.02
	PRAIS	0.02	0.17	0.00	0.02	0.00	0.02	0.00	0.01	0.00	0.02
	PRASCSi	0.02	0.17	0.00	0.02	0.00	0.02	0.00	0.01	0.00	0.02
	PRFGS	0.02	0.17	0.00	0.02	0.00	0.02	0.00	0.01	0.00	0.02
	PSCVT	0.02	0.20	0.00	0.02	0.00	0.02	0.00	0.01	0.00	0.02
	PSP	0.08	0.74	0.01	0.06	0.01	0.06	0.23	1.54	0.01	0.07
	PTAr	0.30	2.75	0.03	0.29	0.54	4.66	1.26	8.47	1.15	9.65
	QULNS	0.00	0.00	0.00	0.00	0.00	0.00	0.00	0.00	0.00	0.00
	RBFSa	0.00	0.00	0.00	0.00	0.00	0.00	0.00	0.00	0.00	0.00
	SBP	0.11	1.04	0.00	0.00	0.87	7.44	1.38	9.27	0.45	3.75
	SUCOAS	0.01	0.11	0.00	0.01	0.00	0.01	0.00	0.01	0.00	0.01
	SULabcpp	0.01	0.10	0.00	0.01	0.00	0.01	0.00	0.01	0.00	0.01
	THRS	0.03	0.28	0.00	0.03	0.00	0.03	0.00	0.02	0.00	0.03
	UAAGDS	0.00	0.01	0.00	0.00	0.00	0.00	0.00	0.00	0.00	0.00
	UAGCVT	0.00	0.01	0.00	0.00	0.00	0.00	0.00	0.00	0.00	0.00
	UAMAGS	0.00	0.01	0.00	0.00	0.00	0.00	0.00	0.00	0.00	0.00
	UAMAS	0.00	0.01	0.00	0.00	0.00	0.00	0.00	0.00	0.00	0.00
	UDCPDP	0.00	0.01	0.00	0.00	0.00	0.00	0.00	0.00	0.00	0.00
	UGMDDS	0.00	0.01	0.00	0.00	0.00	0.00	0.00	0.00	0.00	0.00
	ZNabcpp	0.00	0.00	0.00	0.00	0.00	0.00	0.00	0.00	0.00	0.00
	GAPDi_nadp	0.00	0.00	0.45	4.30	3.04	26.12	4.89	32.83	0.34	2.83
	NGAM	0.00	0.00	7.96	75.59	0.00	0.00	0.00	0.00	0.00	0.00
	FBP	0.00	0.00	0.00	0.00	0.49	4.21	1.00	6.74	0.07	0.59
	Isobutene_spontaneous	0.00	0.00	0.00	0.00	0.51	4.41	0.00	0.00	0.00	0.00
	PPS	0.00	0.00	0.00	0.00	0.00	0.00	0.58	3.92	0.00	0.00

Supplementary Table 12 Predicted flux distributions of Pi-consuming reactions in *Synechocystis* simulated to grow autotrophically and mixotrophically, when maximizing biomass and alkenes production. Presented are percentage (%) and the corresponding flux (mmol/gDW/h) of reactions contributing to the consumption of Pi. Zero flux indicates that the reaction carries no flux or less than 0.01 mmol/gDW/h. For the complete list of reactions, including their abbreviations, subsystem, lower and upper bounds, and stoichiometry, refer to data availability section. Auto, autotrophic; Mixo, mixotrophic.

		Biomass		Isoprene		Isobutene		Ethylene		1-undecene	
**trophy**	**reaction**	**flux**	**percent**	**flux**	**percent**	**flux**	**percent**	**flux**	**percent**	**flux**	**percent**
**Auto**	**ATPSu**	-16.04	96.63	-16.19	96.68	-15.84	95.57	-15.04	91.99	-14.46	88.70
	**PKETF**	-0.43	2.57	-0.04	0.26	-0.72	4.35	-1.02	6.22	-1.84	11.30
	**ATPS4rpp_1**	-0.13	0.78	-0.51	3.07	-0.01	0.08	-0.29	1.78	0.00	0.00
	**PUNP1**	0.00	0.01	0.00	0.00	0.00	0.00	0.00	0.00	0.00	0.00
	**MTAP**	0.00	0.00	0.00	0.00	0.00	0.00	0.00	0.00	0.00	0.00
**Mixo**	**ATPSu**	-10.70	97.34	-10.50	99.72	-11.10	95.34	-13.44	90.30	-10.76	90.36
	**PKETX**	-0.15	1.40	0.00	0.00	0.00	0.00	0.00	0.00	-0.70	5.87
	**PKETF**	-0.14	1.25	-0.03	0.28	-0.54	4.66	-1.26	8.47	-0.45	3.77
	**PUNP1**	0.00	0.01	0.00	0.00	0.00	0.00	0.00	0.00	0.00	0.00
	**MTAP**	0.00	0.00	0.00	0.00	0.00	0.00	0.00	0.00	0.00	0.00
	**ATPS4rpp_1**	0.00	0.00	0.00	0.00	0.00	0.00	-0.18	1.23	0.00	0.00

Supplementary Table 13 Predicted flux distributions of pyruvate-producing reactions in *Synechocystis* simulated to grow autotrophically and mixotrophically, when maximizing biomass and alkenes production. Presented are percentage (%) and the corresponding flux (mmol/gDW/h) of reactions contributing to the generation of pyruvate. Zero flux indicates that the reaction carries no flux or less than 0.01 mmol/gDW/h. For the complete list of reactions, including their abbreviations, subsystem, lower and upper bounds, and stoichiometry, refer to data availability section. Auto, autotrophic; Mixo, mixotrophic.

		Biomass		Isoprene		Isobutene		Ethylene		1-undecene	
**trophy**	**reaction**	**flux**	**percent**	**flux**	**percent**	**flux**	**percent**	**flux**	**percent**	**flux**	**percent**
**Auto**	**ADCL**	7E-05	3E-02	7E-06	1E-03	7E-06	5E-04	7E-06	3E-02	7E-06	3E-02
	**AGTi**	7E-05	3E-02	7E-06	1E-03	0E+00	0E+00	0E+00	0E+00	0E+00	0E+00
	**ANS**	6E-03	3E+00	6E-04	8E-02	6E-04	4E-02	6E-04	3E+00	6E-04	3E+00
	**PYK**	1E-01	6E+01	1E-02	2E+00	1E+00	1E+02	0E+00	0E+00	6E-03	3E+01
	**PYK2**	5E-02	2E+01	5E-03	7E-01	5E-03	3E-01	0E+00	0E+00	5E-03	2E+01
	**PYK3**	3E-02	1E+01	3E-03	5E-01	3E-03	2E-01	0E+00	0E+00	3E-03	1E+01
	**SHCHCS2**	1E-04	6E-02	1E-05	2E-03	1E-05	1E-03	1E-05	6E-02	1E-05	6E-02
	**PYK4**	0E+00	0E+00	7E-01	1E+02	1E-03	1E-01	0E+00	0E+00	1E-03	6E+00
	**ME2**	0E+00	0E+00	0E+00	0E+00	0E+00	0E+00	2E-02	1E+02	7E-03	3E+01
**Mixo**	**ADCL**	5E-05	3E-02	5E-06	1E-03	5E-06	5E-04	5E-06	8E-04	5E-06	3E-02
	**ANS**	4E-03	3E+00	4E-04	9E-02	4E-04	4E-02	4E-04	7E-02	4E-04	3E+00
	**EDA**	9E-02	6E+01	8E-02	2E+01	0E+00	0E+00	0E+00	0E+00	0E+00	0E+00
	**ME2**	5E-02	3E+01	5E-03	1E+00	5E-01	5E+01	4E-01	6E+01	5E-03	3E+01
	**SERD_L**	2E-02	1E+01	0E+00	0E+00	0E+00	0E+00	2E-01	4E+01	0E+00	0E+00
	**SHCHCS2**	9E-05	6E-02	9E-06	2E-03	9E-06	9E-04	9E-06	2E-03	9E-06	6E-02
	**PYK4**	0E+00	0E+00	3E-01	8E+01	1E-03	9E-02	0E+00	0E+00	1E-03	6E+00

	PYK	0E+00	0E+00	0E+00	0E+00	6E-01	5E+01	0E+00	0E+00	4E-03	3E+01
	PYK2	0E+00	0E+00	0E+00	0E+00	3E-03	3E-01	0E+00	0E+00	3E-03	2E+01
	PYK3	0E+00	0E+00	0E+00	0E+00	2E-03	2E-01	0E+00	0E+00	2E-03	1E+01

References

1. Zhang, S. & Bryant, D. A. The Tricarboxylic Acid Cycle in Cyanobacteria. *Science (80-.).* **334**, 1551–1553 (2011).

2. Steinhauser, D., Fernie, A. R. & Araújo, W. L. Unusual cyanobacterial TCA cycles: not broken just different. *Trends Plant Sci.* **17**, 503–509 (2012).

3. Xiong, W., Brune, D. & Vermaas, W. F. J. The γ-aminobutyric acid shunt contributes to closing the tricarboxylic acid cycle in *Synechocystis* sp. PCC 6803. *Mol. Microbiol.* **93**, 786–796 (2014).

4. Xiong, W. *et al.* Phosphoketolase pathway contributes to carbon metabolism in cyanobacteria. *Nat. Plants* **2**, 15187 (2016).

5. Bachhar, A. & Jablonsky, J. A new insight into role of phosphoketolase pathway in *Synechocystis* sp. PCC 6803. *Sci. Rep.* **10**, 22018 (2020).

6. Chen, X. *et al.* The Entner–Doudoroff pathway is an overlooked glycolytic route in cyanobacteria and plants. *Proc. Natl. Acad. Sci.* **113**, 5441–5446 (2016).

7. Klemke, F. *et al.* Identification of the light-independent phosphoserine pathway as an additional source of serine in the cyanobacterium *Synechocystis* sp. PCC 6803. *Microbiology* **161**, 1050–1060 (2015).

8. Bonner, C. A., Jensen, R. A., Gander, J. E. & Keyhani, N. O. A core catalytic domain of the TyrA protein family: arogenate dehydrogenase from *Synechocystis*. *Biochem. J.* **382**, 279–291 (2004).

9. Joshi, C. J., Peebles, C. A. M. & Prasad, A. Modeling and analysis of flux distribution and bioproduct formation in *Synechocystis* sp. PCC 6803 using a new genome-scale metabolic reconstruction. *Algal Res.* **27**, 295–310 (2017).

10. Kämäräinen, J. *et al.* Pyridine nucleotide transhydrogenase PntAB is essential for optimal growth and photosynthetic integrity under low-light mixotrophic conditions in *Synechocystis* sp. PCC 6803. *New Phytol.* **214**, 194–204 (2017).

11. Lea-Smith, D. J., Bombelli, P., Vasudevan, R. & Howe, C. J. Photosynthetic, respiratory and extracellular electron transport pathways in cyanobacteria. *Biochim. Biophys. Acta - Bioenerg.* **1857**, 247–255 (2016).

12. Cooley, J. W. & Vermaas, W. F. J. Succinate Dehydrogenase and Other Respiratory Pathways in Thylakoid Membranes of *Synechocystis* sp. Strain PCC 6803: Capacity Comparisons and Physiological Function. *J. Bacteriol.* **183**, 4251–4258 (2001).

13. Mustila, H., Kugler, A. & Stensjö, K. Isobutene production in *Synechocystis* sp. PCC 6803 by introducing α-ketoisocaproate dioxygenase from *Rattus norvegicus*. *Metab. Eng. Commun.* **12**, e00163 (2021).

14. Baldwin, J. E. *et al.* 4-Hydroxyphenylpyruvate dioxygenase appears to display α-ketoisocaproate dioxygenase activity in rat liver. *Bioorg. Med. Chem. Lett.* **5**, 1255–

1260 (1995).

15. Rossoni, L., Hall, S. J., Eastham, G., Licence, P. & Stephens, G. The Putative Mevalonate Diphosphate Decarboxylase from *Picrophilus torridus* Is in Reality a Mevalonate-3-Kinase with High Potential for Bioproduction of Isobutene. *Appl. Environ. Microbiol.* **81**, 2625–2634 (2015).

16. Lindberg, P., Park, S. & Melis, A. Engineering a platform for photosynthetic isoprene production in cyanobacteria, using *Synechocystis* as the model organism. *Metab. Eng.* **12**, 70–79 (2010).

17. Ungerer, J. *et al.* Sustained photosynthetic conversion of CO_2 to ethylene in recombinant cyanobacterium *Synechocystis* 6803. *Energy Environ. Sci.* **5**, 8998 (2012).

18. Yunus, I. S. *et al.* Synthetic metabolic pathways for photobiological conversion of CO_2 into hydrocarbon fuel. *Metab. Eng.* **49**, 201–211 (2018).
